# Reticulon 3 deficiency ameliorates post‐myocardial infarction heart failure by alleviating mitochondrial dysfunction and inflammation

**DOI:** 10.1002/mco2.503

**Published:** 2024-02-28

**Authors:** Bingchao Qi, Tiantian Li, Haixia Luo, Lang Hu, Renqian Feng, Di Wang, Tingwei Peng, Gaotong Ren, Dong Guo, Mingchuan Liu, Qiuhe Wang, Mingming Zhang, Yan Li

**Affiliations:** ^1^ Department of Cardiology Tangdu Hospital Air Force Medical University Xi'an Shaanxi China

**Keywords:** heart failure, inflammation, mitochondrial function, myocardial infarction, reticulon 3

## Abstract

Multiple molecular mechanisms are involved in the development of heart failure (HF) after myocardial infarction (MI). However, interventions targeting these pathological processes alone remain clinically ineffective. Therefore, it is essential to identify new therapeutic targets for alleviating cardiac dysfunction after MI. Here, gain‐ and loss‐of‐function approaches were used to investigate the role of reticulon 3 (RTN3) in HF after MI. We found that RTN3 was elevated in the myocardium of patients with HF and mice with MI. Cardiomyocyte‐specific RTN3 overexpression decreased systolic function in mice under physiological conditions and exacerbated the development of HF induced by MI. Conversely, *RTN3* knockout alleviated cardiac dysfunction after MI. Mechanistically, RTN3 bound and mediated heat shock protein beta‐1 (HSPB1) translocation from the cytosol to the endoplasmic reticulum. The reduction of cytosolic HSPB1 was responsible for the elevation of TLR4, which impaired mitochondrial function and promoted inflammation through toll‐like receptor 4 (TLR4)/peroxisome proliferator‐activated receptor gamma coactivator‐1 alpha(PGC‐1α) and TLR4/Nuclear factor‐kappa B(NFκB) pathways, respectively. Furthermore, the HSPB1 inhibitor reversed the protective effect of *RTN3* knockout on MI. Additionally, elevated plasma RTN3 level is associated with decreased cardiac function in patients with acute MI. This study identified RTN3 as a critical driver of HF after MI and suggests targeting RTN3 as a promising therapeutic strategy for MI and related cardiovascular diseases.

## Graphical abstract

Myocardial infarction causes an increase in reticulon 3, which binds to and recruits heat shock protein beta‐1 (HSPB1) into the endoplasmic reticulum, resulting in a decrease in the content of HSPB1 in the cytoplasm. Reduced HSPB1 exacerbates mitochondrial dysfunction and inflammatory responses through the TLR4/PGC‐1α and TLR4/NFκB pathways, respectively, which eventually leads to heart failure.

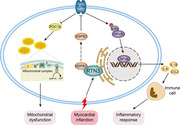

## 1 INTRODUCTION

Myocardial infarction (MI) is the leading cause of morbidity and mortality worldwide.^1^ Because of the extremely limited regenerative capacity of cardiomyocytes, a substantial loss of functional cardiomyocytes is caused by MI, which is the primary pathological process leading to heart failure (HF).^2^, ^3^ Although present advances in percutaneous coronary intervention (PCI) and thrombolysis have yielded better clinical outcomes to some extent, an effective treatment strategy for MI and relief of subsequent HF remain a large unmet medical need.^4^, ^5^ Therefore, there is an urgent need to elucidate the related mechanisms and identify new therapeutic targets to alleviate HF after MI.

MI is an acute injury to cardiomyocytes owing to interruption or reduction of blood flow caused by coronary artery occlusion. Cardiac repair after MI is a complex process involving various pathological mechanisms and signaling pathways. Mitochondrial dysfunction, inflammation, and myocardial fibrosis have long been considered as crucial underlying mechanisms responsible for the development of HF after MI.^6^, ^7^, ^8^ The heart is a metabolically active organ, accounting for only 0.5% of the body weight but consuming nearly 8% of the total adenosine triphosphate(ATP).^9^ In general, 95% of the ATP consumed by the heart is obtained from oxidative phosphorylation along the mitochondrial electron transport chain (ETC). Studies have reported that patients and animal models with MI have compromised cardiac mitochondrial function, which greatly affects HF development.^10^, ^11^ Additionally, after MI occurs, cardiomyocytes and resident immune cells in the heart release inflammatory mediators to recruit immune cells for infiltration.^12^ Activation of early inflammation is essential for cardiac repair. However, because of the cascade effect of the inflammatory response, persistent immune cell infiltration causes severe damage to remaining viable cardiomyocytes and excessive fibroblasts activation, aggravating cardiac dysfunction and myocardial fibrosis.^8^, ^13^ Various mechanisms are involved in post‐MI HF development, and interventions targeting mitochondrial dysfunction or inflammation alone have not been proven clinically effective in alleviating cardiac dysfunction.^14^, ^15^ Therefore, there is an urgent need for new therapeutic targets that can simultaneously alleviate mitochondrial dysfunction and inflammatory responses to further improve cardiac function in patients with MI.

Reticulons (RTNs) are proteins located in the membranes of endoplasmic reticulum (ER). They are characterized by a highly conserved reticulon homology domain (RHD), a 150–200 amino acid C‐terminal sequence that plays an essential role in protein localization and function.^16^ Owing to alternative splicing, RTNs occur in multiple isoforms with different functions.^17^ All RTNs are present in the brain and thus have been primarily studied for axonal regeneration and amyloid deposition.^18^ However, many studies have reported the critical role of these isoforms in ER homeostasis, membrane trafficking, apoptosis, calcium homeostasis, and inflammation.^19^, ^20^, ^21^ In this study, we found that the transcriptional level of *RTN3*, rather than other RTNs, was markedly increased in the myocardium of patients with HF and mice with MI, suggesting the indispensable role of RTN3 in the progression of HF after MI.

The present study aimed to determine the role of RTN3 in myocardial injury. We investigated the pathological and physiological effects of *RTN3* deficiency or overexpression on cardiac function and fibrotic phenotypes in mice. Furthermore, the underlying mechanisms of the effect were explored via transcriptome sequencing and functional experiments. Our findings provide novel insights regarding mitochondrial dysfunction and inflammation during myocardial injury repair and identify RTN3‐mediated signaling as a potential therapeutic target to alleviate HF after MI.

## 2 RESULTS

### 2.1 RTN3 expression is increased in the myocardium of patients with HF and mice with MI

**FIGURE 1:**
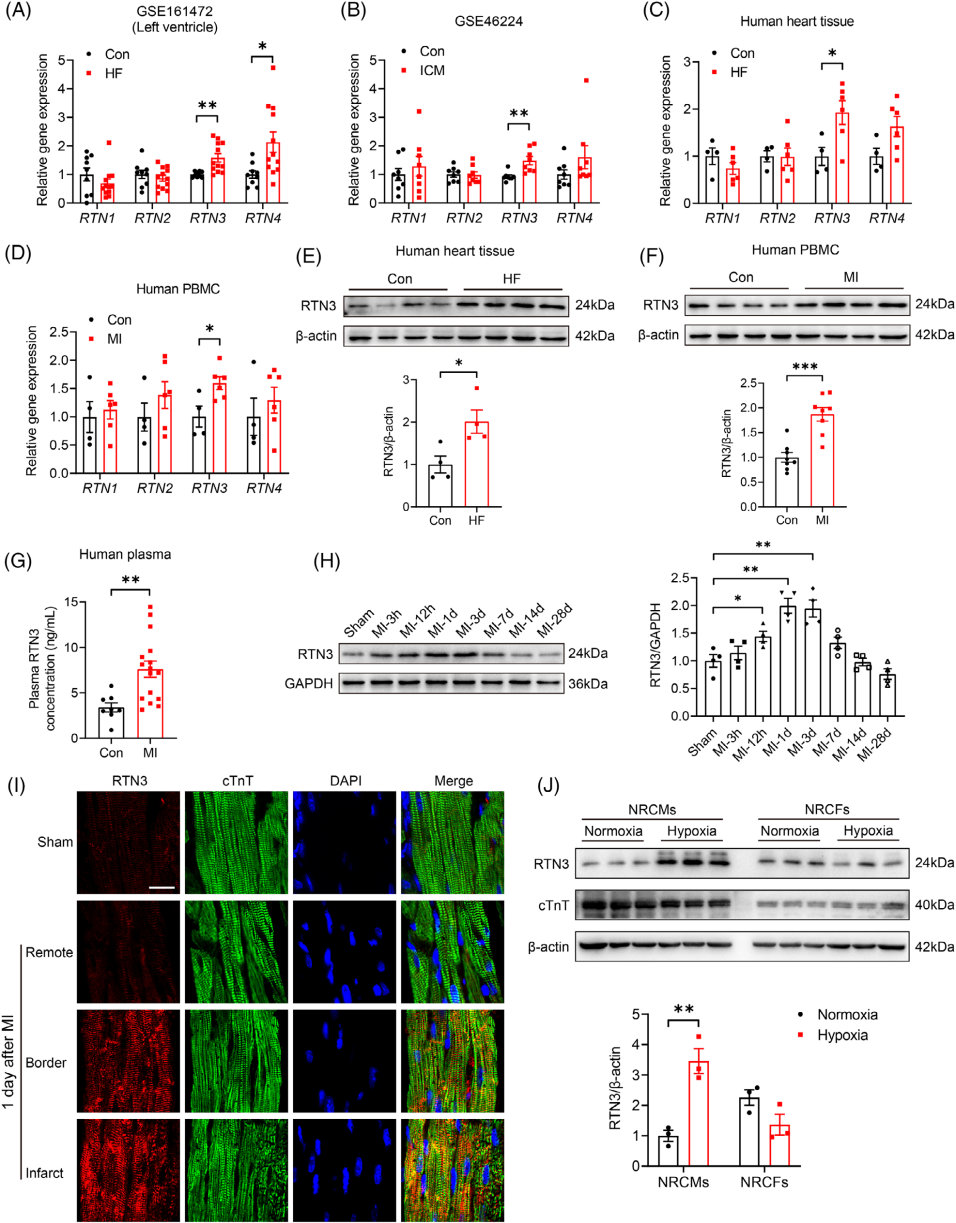
Reticulon 3 (RTN3) expression is increased in the myocardium of patients with heart failure (HF) and mice with myocardial infarction (MI). (A) Transcriptional expression of *RTN1*, *RTN2*, *RTN3*, and *RTN4* in the left ventricle samples RNA sequencing (RNA‐seq) data (GSE161472) including normal subjects (*n* = 9) and patients with HF (*n* = 12). (B) Transcriptional expression of *RTN1*, *RTN2*, *RTN3*, and *RTN4* in human RNA‐seq data (GSE46224), including normal subjects and patients with ischemic cardiomyopathy (ICM) (*n* = 8 per group). (C) Relative mRNA levels of *RTN1*, *RTN2*, *RTN3*, and *RTN4* in hearts of normal subjects (*n* = 4) and patients with HF (*n* = 6). (D) Relative mRNA levels of *RTN1*, *RTN2*, *RTN3*, and *RTN4* in peripheral blood mononuclear cells (PBMCs) of normal subjects (*n* = 4) and patients with MI (*n* = 6). (E) Representative western blots and quantitative results of RTN3 levels in hearts of normal subjects and patients with HF (*n* = 4 per group). (F) Representative western blots and quantitative results of RTN3 levels in PBMCs of normal subjects and patients with MI (*n* = 8 per group). (G) Plasma RTN3 levels in normal subjects (*n* = 8) and patients with MI (*n* = 16). (H) Representative western blots and quantitative analysis of RTN3 protein in mouse hearts at the indicated time points post‐MI (*n* = 4 per group). (I) Immunofluorescence images of RTN3 expression in different areas of mouse hearts 1 day after MI. Cardiac troponin T (cTnT) (green) was used as a cardiomyocyte marker, and 4'‐6‐diamidino‐2‐phenylindole(DAPI, blue) was used to stain the nuclei. Scale bar = 20 μm. (J) Representative western blots and quantitative results of RTN3 levels in neonatal rat primary cardiomyocytes (NRCMs) and neonatal rat cardiac fibroblasts (NRCFs) subjected to hypoxia for 6 h (*n* = 3 per group). Data are presented as mean ± standard error of the mean (SEM). Data in (H) were analyzed by one‐way analysis of variance (ANOVA) with a Bonferroni post hoc test. Others were analyzed by unpaired Student's *t* test. ^*^ *p *< 0.05, ^**^ *p *< 0.01.

To elucidate the transcription profile of the RTNs family in patients with HF, we analyzed two human RNA sequencing (RNA‐seq) datasets. Compared with the control, *RTN3* and *RTN4* expression in the left ventricle of patients with HF was significantly increased (Figure 1A). Ischemic cardiomyopathy (ICM) is one of the important causes of HF. We found that *RTN3* expression was significantly increased in the myocardium of patients with ICM compared with patients without ICM (Figure 1B). Similar to RNA‐seq results, only *RTN3* mRNA expression was higher in the myocardial tissue of patients with HF and peripheral blood mononuclear cells (PBMCs) of patients with MI than those of patients without HF or MI, respectively (Figure 1C,D). Furthermore, detection at the protein level confirmed that RTN3 was markedly increased in the myocardium of patients with HF and PBMCs of patients with MI (Figure 1E,F). In addition, plasma RTN3 levels in patients with MI were notably increased compared with patients without MI (Figure 1G). We constructed a MI mouse model to further investigate the temporal and spatial specificities of RTN3 expression in the myocardium after MI. The expression of RTN3 protein began to increase at 12 h post‐MI, reached a maximum value of 2.0‐fold at 1–3 days post‐MI, and then gradually decreased to the normal levels (Figure 1H). The immunofluorescence assay confirmed that MI‐induced increase in RTN3 level was mainly in the infarct region and infarct border zone rather than the remote zone (Figure 1I). To further confirm the origin of the increased RTN3 level in the heart, we treated two major primary cell types with hypoxia. Hypoxia‐induced increase in RTN3 level was observed in neonatal rat primary cardiomyocytes (NRCMs) but not in neonatal rat cardiac fibroblasts (Figure 1J). Based on this MI‐ and hypoxia‐induced marked increase in RTN3 level, we speculate the irrefutable involvement of RTN3 in MI and HF pathogenesis.

### 2.2 Cardiomyocyte‐specific RTN3 overexpression aggravates cardiac dysfunction after MI, while *RTN3* knockout alleviates post‐MI heart failure

To explore the potential effect of RTN3 on cardiac function, an adeno‐associated virus (AAV) with a cardiomyocyte‐specific promoter, cardiac troponin T (cTnT), was constructed and injected into the thoracic cavity of C57BL/6J mice (Figure S1A). The mRNA and protein levels of RTN3 were increased in mice injected with AAV serotype 9 encoding mouse *RTN3* (AAV9‐*RTN3*) for 3 weeks (Figure S1B,C). The immunofluorescence assay revealed that increased RTN3 levels in the heart were mainly located in the left ventricular (LV) region (Figure S1D). Additionally, AAV9‐mediated increase in RTN3 level only occurred in the cardiac tissue but not in the other organs, facilitating the evaluation of the effect of RTN3 on cardiac function (Figure S1E). The timeline of the experimental design of this study is illustrated in Figure S2A. RTN3 overexpression for 12 weeks increased heart weight; however, there was no change in lung weight under physiological conditions (Figure S2B). Echocardiography revealed that RTN3 overexpression triggered a notable decrease in systolic function and LV dilatation, as evidenced by decreased ejection fraction (EF) and fractional shortening (FS) and increased left ventricular internal dimension at systolic phase (LVIDs) and left ventricular internal dimension at diastolic phase (LVIDd), respectively (Figure S2C). Compared with the control group, the hearts of the RTN3 overexpression group exhibited dramatically increased perivascular and interstitial fibroses (Figure S2D). Furthermore, HF markers such as atrial natriuretic peptide (ANP) and brain natriuretic peptide (BNP) and fibrosis markers (Col1a1 and Col3a1) were significantly elevated in the hearts of mice injected with AAV9‐*RTN3* for 12 weeks (Figure S2E). Taken together, these results suggest that RTN3 overexpression via a gain‐of‐function approach drives the onset of cardiac dysfunction and cardiac fibrosis under physiological conditions.

**FIGURE 2:**
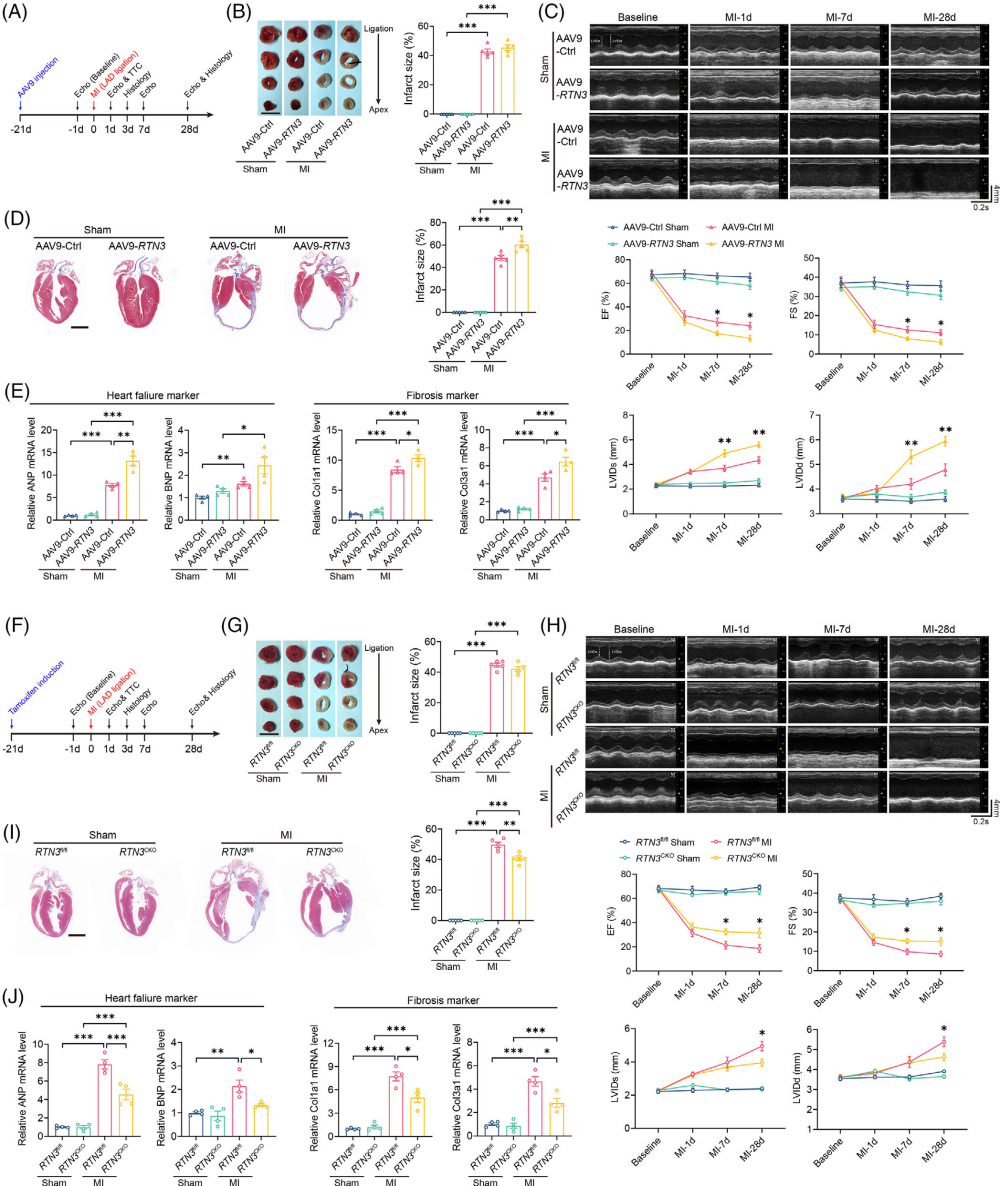
Cardiomyocyte‐specific reticulon 3 (RTN3) overexpression aggravates cardiac dysfunction and *RTN3* knockout alleviates heart failure (HF) after myocardial infarction (MI). (A) The timeline of the experimental design for RTN3 overexpression in mice after MI. (B) Infarct size was evaluated by triphenyl tetrazolium chloride (TTC) staining 24 h post‐MI (*n* = 5 per group). Scale bar = 5 mm. (C) Representative M‐mode echocardiography images at the indicated time points post‐MI. Echocardiography parameters, including ejection fraction (EF), fractional shortening (FS), left ventricular internal dimension at systolic phase (LVIDs), and left ventricular internal dimension at diastolic phase (LVIDd), were calculated (*n* = 8 per group). (D) Representative Masson trichrome staining images and quantification of infarct size 28 days post‐MI (*n* = 5 per group). Scale bar = 2 mm. (E) Relative mRNA levels of ANP, BNP, Col1a1, and Col3a1 in hearts 28 days post‐MI (*n* = 4 per group). (F) The timeline of the experimental design for *RTN3* knockout in mice after MI. (G) Infarct size was evaluated by TTC staining 24 h post‐MI (*n* = 5 per group). Scale bar = 5 mm. (H) Representative M‐mode echocardiography images at the indicated time points post‐MI. Echocardiography parameters, including EF, FS, LVIDs, and LVIDd, were calculated (*n* = 8 per group). (I) Representative Masson trichrome staining images and quantification of infarct size 28 days post‐MI (*n* = 5 per group). Scale bar = 2 mm. (J) Relative mRNA levels of ANP, BNP, Col1a1, and Col3a1 in hearts 28 days post‐MI (*n* = 4 per group). Data are presented as mean ± standard error of the mean (SEM). Statistical significance was assessed by one‐way analysis of variance (ANOVA) with a Bonferroni post hoc test. ^*^ *p *< 0.05, ^**^ *p *< 0.01, ^***^ *p *< 0.001.

To assess the effect of RTN3 overexpression on cardiac function under pathological conditions, we constructed a mouse MI model by permanently ligating the left anterior descending (LAD) coronary artery (Figure 2A). At 24 h post‐MI, triphenyl tetrazolium chloride staining showed that the MI group had an obvious infarction zone, and the infarct size was comparable between the AAV9‐Ctrl and AAV9‐*RTN3* mice post‐MI (Figure 2B). Echocardiography at different time points revealed a sharp decline in the post‐MI cardiac function compared with the sham group, whereas RTN3 overexpression significantly aggravated MI‐induced systolic dysfunction and LV dilatation (Figure 2C). At 28 days post‐MI, Masson staining revealed that AAV9‐*RTN3* mice had a larger infarct area than AAV9‐Ctrl mice (Figure 2D). Additionally, the mRNA expression of HF and cardiac fibrosis markers was increased in the hearts of mice in the AAV9‐*RTN3* group compared with those in the AAV9‐Ctrl group (Figure 2E). Taken together, these results suggest that cardiomyocyte‐specific RTN3 overexpression exacerbates MI‐induced cardiac dysfunction and remodeling.

In addition to gain‐of‐function experiments, loss‐of‐function experiments were performed using cardiomyocyte‐specific *RTN3* knockout mice via the Cre/loxP‐dependent conditional gene knockout approach (Figure S3A). The genotypes of *RTN3* knockout mice (*RTN3* ^CKO^) and strict littermate control mice (*RTN3* ^fl/fl^) were identified using agarose gel electrophoresis (Figure S3B). The mRNA and protein of RTN3 were efficiently knocked out in the myocardium of *RTN3* ^CKO^ mice after tamoxifen induction (Figure S3C,D). Next, we elucidated the effects of *RTN3* deficiency on the cardiac function and fibrotic phenotypes of mice under pathological and physiological conditions (Figures 2F and S4A). Interestingly, there were no significant differences in the heart and lung weight of *RTN3* ^CKO^ mice and control mice (Figure S4B). Under physiological conditions, cardiomyocyte‐specific knockout of *RTN3* did not significantly affect the systolic function and LV diameter of mice, as demonstrated by similar EF, FS, LVIDs, and LVIDd via echocardiography for 12 consecutive weeks (Figure S4C). There was no significant difference in the infarct size between *RTN3* ^fl/fl^ and *RTN3* ^CKO^ mice 24 h after MI (Figure 2G); however, compared with AAV9‐Ctrl mice subjected to MI, *RTN3* deficiency noticeably alleviated cardiac dysfunction and LV dilatation at 28 days post‐MI (Figure 2H). Under physiological conditions, myocardial perivascular and interstitial fibroses were not observed in both groups at 12 weeks after tamoxifen induction (Figure S4D). Mice with MI exhibited a large infarct area; however, *RTN3* knockout significantly decreased the infarct size (Figure 2I). Furthermore, under physiological conditions, HF and fibrosis markers were roughly comparable in the hearts of *RTN3* ^CKO^ and *RTN3* ^fl/fl^ mice (Figure S4E). MI surgery dramatically increased the levels of HF and fibrosis markers in the hearts of *RTN3* ^fl/fl^ mice, whereas these changes were rescued in the hearts of *RTN3* ^CKO^ mice (Figure 2J). Together, these in vivo results suggest that decreasing the abundance of RTN3 may provide an effective strategy to protect the heart from post‐MI cardiac fibrosis and HF.

### 2.3 *RTN3* deficiency rescues mitochondrial ETC dysfunction in the myocardium after MI

**FIGURE 3:**
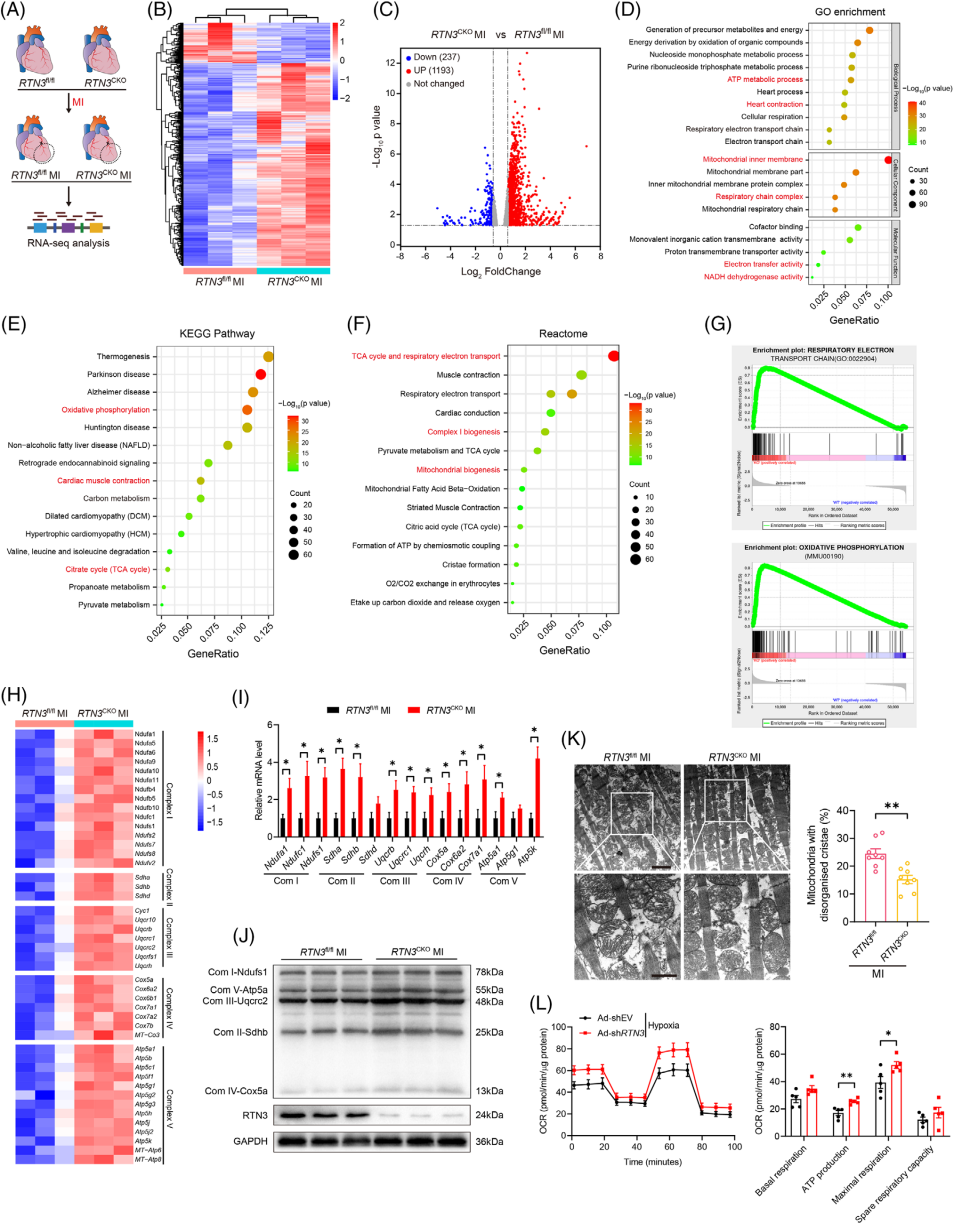
Reticulon 3 (*RTN3*) deficiency rescues mitochondrial electron transport chain (ETC) dysfunction in the myocardium after myocardial infarction (MI). (A) Schematic diagram showing the experimental design of RNA sequencing (RNA‐seq). (B) Hierarchical cluster analysis of differentially expressed genes (DEGs) in *RTN3* ^CKO^ versus *RTN3* ^fl/fl^ mice post‐MI as assessed by RNA‐seq (fold change > 1.5, *p *< 0.05). (C) Volcano plot of DEGs in indicated mouse hearts (fold change > 1.5, *p *< 0.05). (D–F) Gene Ontology (GO), Kyoto Encyclopedia of Genes and Genomes (KEGG), and Reactome enrichment analysis of DEGs in indicated mouse hearts using the Database for Annotation and Visualization and Integrated Discovery (DAVID) tools. (G) Gene Set Enrichment Analysis (GSEA) analysis of metabolic‐related enrichment plots which upregulated in *RTN3* ^CKO^ mice compared with *RTN3* ^fl/fl^ mice after MI. (H) Heatmap showing differentially expressed mitochondrial ETC complex subunit genes in RNA‐seq data. (I) Quantitative polymerase chain reaction (PCR) analysis of mRNA levels of mitochondrial ETC complex subunit genes (*n* = 5 per group). (J) Western blots images of mitochondrial ETC complex subunits in indicated groups (*n* = 3 per group). (K) Representative transmission electron microscopy images and quantification of abnormal mitochondria in mice post‐MI (*n* = 8 per group). The upper scale bar = 2 μm and the lower scale bar = 1 μm. (L) oxygen consumption rate (OCR) detection and quantitative analysis in neonatal rat primary cardiomyocytes (NRCMs) with or without *RTN3* knockdown after hypoxia stimulation (*n* = 5 per group). Data are presented as mean ± standard error of the mean (SEM). Statistical significance was assessed by unpaired Student's *t* test. ^*^ *p *< 0.05, ^**^ *p *< 0.01.

To determine the mechanisms by which *RTN3* knockout improves post‐MI HF, we performed unbiased RNA‐seq of LV tissues from *RTN3* ^CKO^ and *RTN3* ^fl/fl^ mice 3 days post‐MI (Figure 3A). Hierarchical clustering analysis displayed that *RTN3* knockout markedly altered the cardiac transcriptome profile post‐MI (Figure 3B). Furthermore, volcano plot analysis identified 1430 differentially expressed genes (DEGs) between the two groups, with 237 downregulated and 1193 upregulated genes (Figure 3C). The Database for Annotation and Visualization and Integrated Discovery was used to determine the functional annotation of these DEGs. Gene Ontology analysis revealed that the DEGs were closely associated with myocardial energy metabolism, involving biological processes such as “ATP metabolic process” and “heart contraction,” cellular components such as “mitochondrial inner membrane” and “respiratory chain complex,” and molecular functions such as “electron transfer activity” and “NADH dehydrogenase activity” (Figure 3D). Similarly, Kyoto Encyclopedia of Genes and Genomes and Reactome pathway analyses further revealed that *RTN3* knockout altered the mitochondrial signaling pathways and biological responses, including “oxidative phosphorylation,” “cardiac muscle contraction,” “carbon metabolism,” “The tricarboxylic acid (TCA) cycle and respiratory electron transport,” and “mitochondrial biogenesis” (Figure 3E,F). These enrichment analyses suggested that the improvement of *RTN3* knockout on HF after MI may be mediated by mitochondrial energy metabolism. Gene Set Enrichment Analysis demonstrated that “respiratory electron transport chain” and “oxidative phosphorylation” were markedly upregulated in *RTN3* ^CKO^ mice compared with *RTN3* ^fl/fl^ mice (Figure 3G).

The mitochondrial ETC contains five complexes and is the primary site of ATP production via oxidative phosphorylation in the cardiomyocytes. Consistent with the findings of enrichment analysis, genes related to the subunits of the mitochondrial complexes were upregulated in the hearts of *RTN3* knockout mice (Figure 3H). To verify these results, mRNA and protein expression levels of the ETC complex subunits were subsequently determined. As expected, the expression of representative genes in the ETC complex subunits was significantly increased in *RTN3* knockout mice compared with control mice after MI (Figure 3I,J). Changes in mitochondrial ETC complexes are often accompanied by aberrations in the mitochondrial morphology and respiratory dysfunction. Therefore, we assessed the mitochondrial ultrastructure under a transmission electron microscope (TEM) and observed that the hearts of mice with MI exhibited abnormal mitochondria with disarrayed cristae and decreased electron density; these anomalies were alleviated in the hearts of *RTN3* ^CKO^ mice (Figure 3K). Furthermore, to confirm the effect of RTN3 on mitochondrial respiration, we constructed RTN3 upregulating or downregulating adenoviruses and verified their effects at the mRNA and protein levels (Figure S5). Consistent with the in vivo results, *RTN3* knockdown in NRCMs significantly increased the mitochondrial respiratory capacity, including ATP production‐coupled respiration and maximal respiration, under hypoxic conditions (Figure 3L). Collectively, these data indicate that *RTN3* deficiency alleviates cardiac dysfunction post‐MI by improving mitochondrial respiration.

### 2.4 RTN3 binds to heat shock protein beta‐1 and recruits it to the ER

**FIGURE 4:**
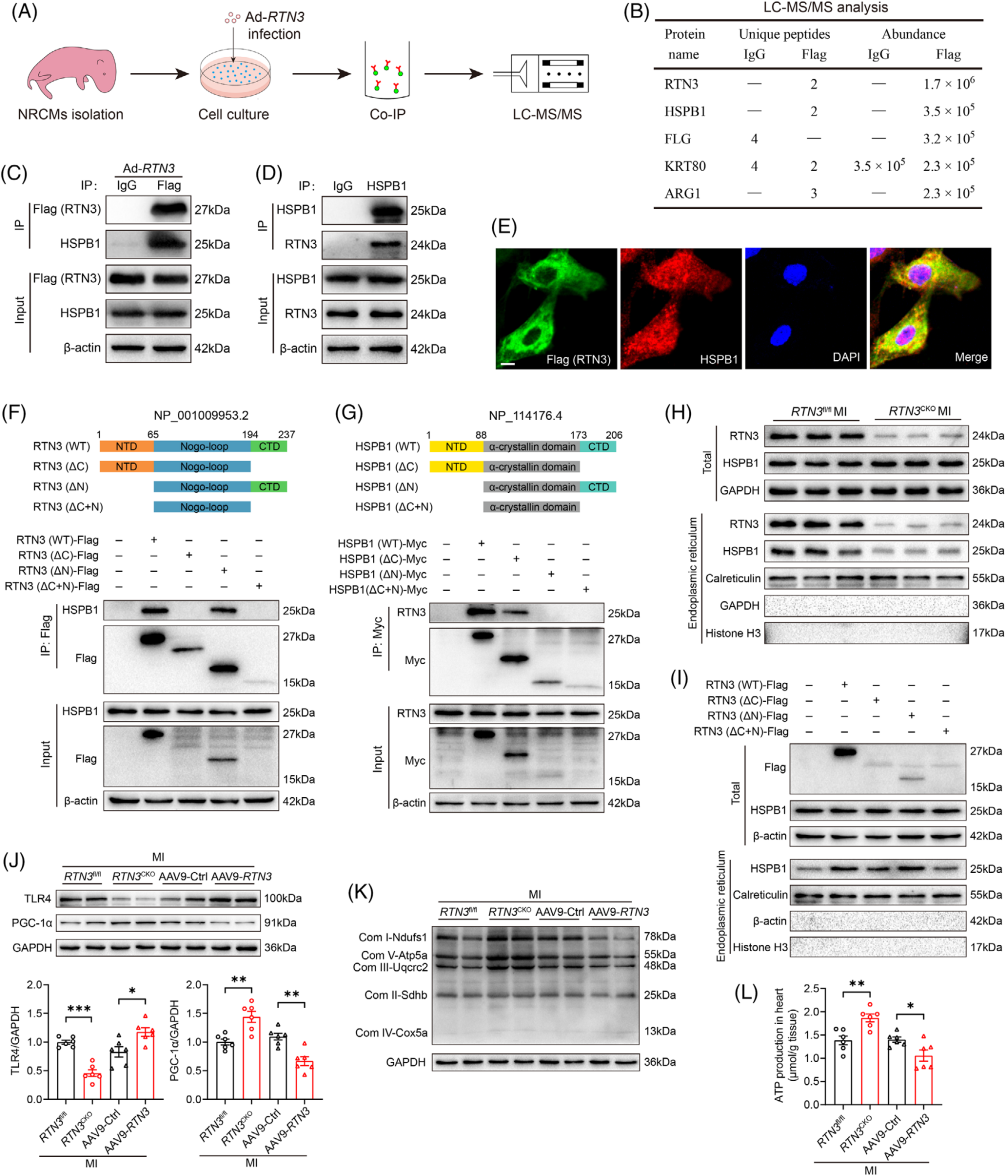
Reticulon 3 (RTN3) binds to heat shock protein beta‐1 (HSPB1) and recruits it to the endoplasmic reticulum. (A) Schematic diagram showing the experimental design of screening interacting proteins. (B) Mass spectrometry analysis of immunoprecipitated results. (C and D) Representative western blots showing the interaction of RTN3 and HSPB1 using anti‐Flag and anti‐HSPB1 antibodies in neonatal rat primary cardiomyocytes (NRCMs), respectively. (E) Representative immunofluorescence images showing co‐location for RTN3 (green) and HSPB1 (red) in NRCMs. DAPI (blue) was used to stain the nuclei. Scale bar = 10 μm. (F) The upper: schematic diagram showing the construction of RTN3 structure and its truncated forms; the lower: H9c2 cells were transfected with wild‐type RTN3 or its truncated mutants and applied to immunoprecipitation (IP) assay. CTD, C‐terminal domain; NTD, N‐terminal domain. (G) The upper: schematic diagram showing the construction of HSPB1 structure and its truncated forms; the lower: H9c2 cells were transfected with wild‐type HSPB1 or its truncated mutants and applied to IP assay. (H) Representative western blots of endoplasmic reticulum (ER) proteins in the hearts of *RTN3* ^CKO^ mice and *RTN3* ^fl/fl^ mice after MI. Calreticulin was used as an internal reference for ER proteins, and Histone H3 was used as an internal reference for nuclear proteins. (I) H9c2 cells were transfected with indicated plasmids, and ER fractions were extracted for western blot. (J) Western blot analysis of TLR4 and PGC‐1α expression in the hearts of indicated mice (*n* = 6 per group). (K) Representative western blots of mitochondrial electron transport chain (ETC) complex subunits in indicated groups. (L) ATP production in the hearts of indicated mice (*n* = 6 per group). Data are presented as mean ± standard error of the mean (SEM). Data were analyzed by unpaired Student's *t* test. ^*^ *p *< 0.05, ^**^ *p *< 0.01, ^***^ *p *< 0.001.

Considering the crucial role of the mitochondrial ETC complexes in HF progression, we investigated the potential mechanisms by which *RTN3* knockout improves mitochondrial respiration. First, NRCMs were infected with Flag‐tagged adenovirus overexpressing RTN3; then co‐immunoprecipitation (Co‐IP) and mass spectrometry were performed to screen the interacting proteins (Figure 4A). The top five most abundant proteins were identified using mass spectrometry (Figure 4B). Next, the Co‐IP assay was respectively performed using the Flag antibody and heat shock protein beta‐1 (HSPB1) antibody, confirming that RTN3 directly interacted with HSPB1 in NRCMs (Figure 4C,D). Moreover, the immunofluorescence assay revealed co‐localization of RTN3 and HSPB1 in the cardiomyocytes (Figure 4E). Because RTN3 is a transmembrane protein with N‐ and C‐terminal domains facing the cytoplasmic compartment, we constructed truncated mutants to identify the molecular domains involved in this interaction (Figure 4F). The Co‐IP assays further revealed that deletion of the C‐terminal domain, but not the N‐terminal domain, prevented the interaction between RTN3 and HSPB1 (Figure 4F). Similarly, HSPB1 truncated mutant plasmids were constructed and transfected into H9c2 cells for the Co‐IP assays (Figure 4G). RTN3 interacted with wild‐type HSPB1 and the HSPB1 (ΔC) domain but not with the HSPB1 (ΔN) and HSPB1 (ΔC + N) domain; this finding suggests that the N‐terminal domain of HSPB1 is essential for the interaction between RTN3 and HSPB1 (Figure 4G). In addition, compared with control mice with MI, *RTN3* knockout did not affect the total amount of HSPB1 protein but markedly reduced the amount of HSPB1 in the ER (Figure 4H). Furthermore, wild‐type RTN3 and RTN3 (ΔN) mutants enhanced the localization of HSPB1 in the ER, whereas this phenomenon was not observed in RTN3 (ΔC) mutant‐transfected H9c2 cells (Figure 4I). Taken together, the results suggest that although RTN3 does not affect the total amount of HSPB1 in the cell, it can recruit HSPB1 to the ER, thereby reducing the relative content of HSPB1 in the cytoplasm.

### 2.5 RTN3‐mediated HSPB1 translocation respectively regulates post‐MI mitochondrial biogenesis and inflammation via the TLR4/PGC‐1α pathway and TLR4/NFκB pathway

Given that *RTN3* knockout caused marked upregulation of mitochondrial ETC genes, the mitochondrial biogenesis pathway attracted our attention. First, heatmap clustering analysis of the RNA‐seq data revealed that the transcriptional coactivators *PGC‐1α* and *PGC‐1β* were significantly elevated in the hearts of *RTN3* knockout mice (Figure S6A). Next, we performed Pearson correlation analysis of the sequencing data from human myocardium and found that *PGC‐1α*, but not *PGC‐1β*, was negatively correlated with RTN3; this suggests that RTN3 negatively regulates PGC‐1α expression (Figure S6B). Surgical induction of MI dramatically decreased PGC‐1α protein level in the hearts of *RTN3* ^fl/fl^ mice; nevertheless, this change was partly rescued in the hearts of *RTN3* ^CKO^ mice (Figure S6C). Previous studies have reported that TLR4 regulates PGC‐1α after myocardial ischemia and that TLR4 abundance is associated with HSPB1 level.^22^, ^23^ As a result, we knocked down *TLR4* and *HSPB1*, respectively, in vitro to verify the relationship among PGC‐1α, TLR4, and HSPB1. We found that HSPB1 knockdown increased TLR4 expression and decreased PGC‐1α expression, which could be prevented via *TLR4* knockdown (Figure S6D). In addition, HSPB1 overexpression in vitro significantly decreased TLR4 level and increased PGC‐1α level (Figure S6E). These results provide preliminary evidence on the upstream and downstream relationship among PGC‐1α, TLR4, and HSPB1.

Next, we isolated ER components from the myocardium of mice to investigate the effect of RTN3 on downstream genes. In vivo, upregulation or downregulation of RTN3 did not significantly affect the total amount of HSPB1 in the MI mice (Figure S6F). However, *RTN3* knockout significantly reduced HSPB1 expression in the ER and increased the content in the cytoplasm, while the overexpression of RTN3 resulted in the opposite effect (Figure S6F). This relative decrease of HSPB1 in the cytoplasm possibly affected TLR4 levels. As expected, *RTN3* knockout decreased TLR4 expression and increased PGC‐1α expression in the myocardium post‐MI, whereas AAV9‐mediated RTN3 upregulation significantly increased TLR4 expression and decreased PGC‐1α expression (Figure 4J). PGC‐1α is well‐known as the key regulator of mitochondrial biogenesis. Therefore, we determined the effect of RTN3 on critical genes of the mitochondrial ETC complexes. Downregulation of RTN3 increased the protein levels of mitochondrial complex‐related genes after MI; however, its upregulation exerted an opposite effect (Figure 4K). Energy supply plays an essential role in mitochondrial ETC. We observed that ATP production was increased in the hearts of *RTN3* knockout mice and decreased in those of RTN3 overexpressing mice (Figure 4L). Taken together, these observations indicate that the RTN3/HSPB1/TLR4 axis disrupts PGC‐1α expression to drive post‐MI mitochondrial respiratory dysfunction.

**FIGURE 5:**
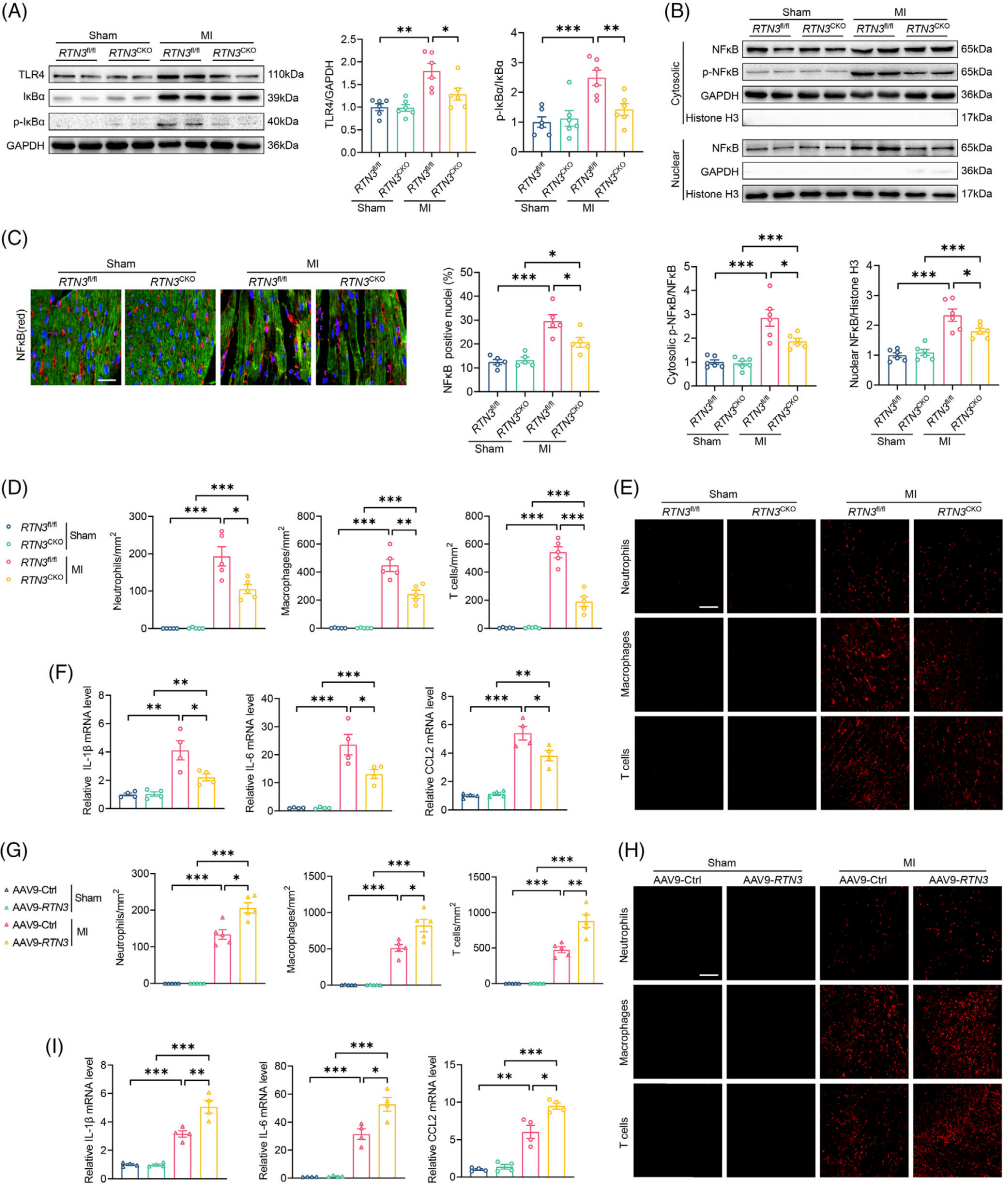
Reticulon 3 (*RTN3*) knockout or overexpression respectively alleviates or aggravates myocardial infarction (MI)‐induced inflammatory response. (A) Representative western blots and quantitative analysis of TLR4 and p‐IκBα in indicated groups (*n* = 6 per group). (B) Representative western blots and quantitative analysis of p‐NFκB or NFκB in cytoplasm and nucleus (*n* = 6 per group). (C) Representative immunofluorescence images and quantitative analysis of NFκB nuclear translocation in indicated groups (*n* = 5 per group). Scale bar = 30 μm. (D) Relative mRNA levels of interleukin (IL)‐1β, IL‐6, and C‐C motif chemokine ligand 2 (CCL2) in hearts of indicated mice 3 days after MI (*n* = 4 per group). (E and F) Representative immunofluorescence images and quantitative analysis of immune cell infiltration in hearts of indicated mice 3 days after MI (*n* = 5 per group). Scale bar = 100 μm. Ly6G, F4/80, and CD3 were used as markers for neutrophils, macrophages, and T cells, respectively. (G) Relative mRNA levels of IL‐1β, IL‐6, and CCL2 in hearts of indicated mice 3 days after MI (*n* = 4 per group). (H and I) Representative immunofluorescence images and quantitative analysis of immune cell infiltration in hearts of indicated mice 3 days after MI (*n* = 5 per group). Scale bar = 100 μm. Data are presented as mean ± standard error of the mean (SEM). Statistical significance was assessed by one‐way analysis of variance (ANOVA) with a Bonferroni post hoc test. ^*^ *p *< 0.05, ^**^ *p *< 0.01, ^***^ *p *< 0.001.

TLR4, a vital member of the pattern recognition receptor family, can induce inflammatory responses after tissue injury. Surgical induction of MI resulted in the elevation of TLR4 expression and p‐IκBα/IκBα level, whereas *RTN3* knockout reversed the inflammatory signals (Figure 5A). Activation of the TLR4/p‐IκBα pathway is essential for NFκB phosphorylation and its translocation into the nucleus. To verify this, we extracted the nuclear components from cardiac tissues and demonstrated that *RTN3* knockout reduced NFκB activation and nuclear translocation to a certain extent (Figure 5B). The immunofluorescence assay revealed a significant decrease in MI‐induced nuclear translocation of NFκB in the hearts of *RTN3* ^CKO^ mice compared with those of *RTN3* ^fl/fl^ mice (Figure 5C).

Furthermore, activation of the TLR4/NFκB pathway could mediate the cardiac resident cells to release inflammatory factors in early‐stage MI and the recruitment of immune cells to the injured area. The cascade amplification effect of the inflammatory signaling pathways can aggravate myocardial injury; therefore, it is important to alleviate the early inflammatory responses of MI. Under physiological conditions, no significant difference was detected in the hearts of *RTN3* ^fl/fl^ and *RTN3* ^CKO^ mice; however, the mRNA levels of inflammatory mediators such as interleukin (IL)‐1β, IL‐6, and CCL2 were decreased in *RTN3* ^CKO^ mice compared with *RTN3* ^fl/fl^ mice after MI (Figure 5D). Consistent with the decrease in inflammatory mediators, neutrophil, macrophage, and T‐cell infiltration were markedly relieved in the hearts of *RTN3* ^CKO^ mice compared with those of *RTN3* ^fl/fl^ post‐MI (Figure 5E,F). In contrast, AAV9‐mediated overexpression of RTN3 significantly increased inflammatory mediator release and immune cell infiltration after MI (Figure 5G–I). These data suggest that RTN3 can regulate inflammatory responses via the TLR4/NFκB signaling pathways under ischemic conditions.

### 2.6 HSPB1 inhibition abolishes the ameliorative effect of *RTN3* knockout on HF after MI

**FIGURE 6:**
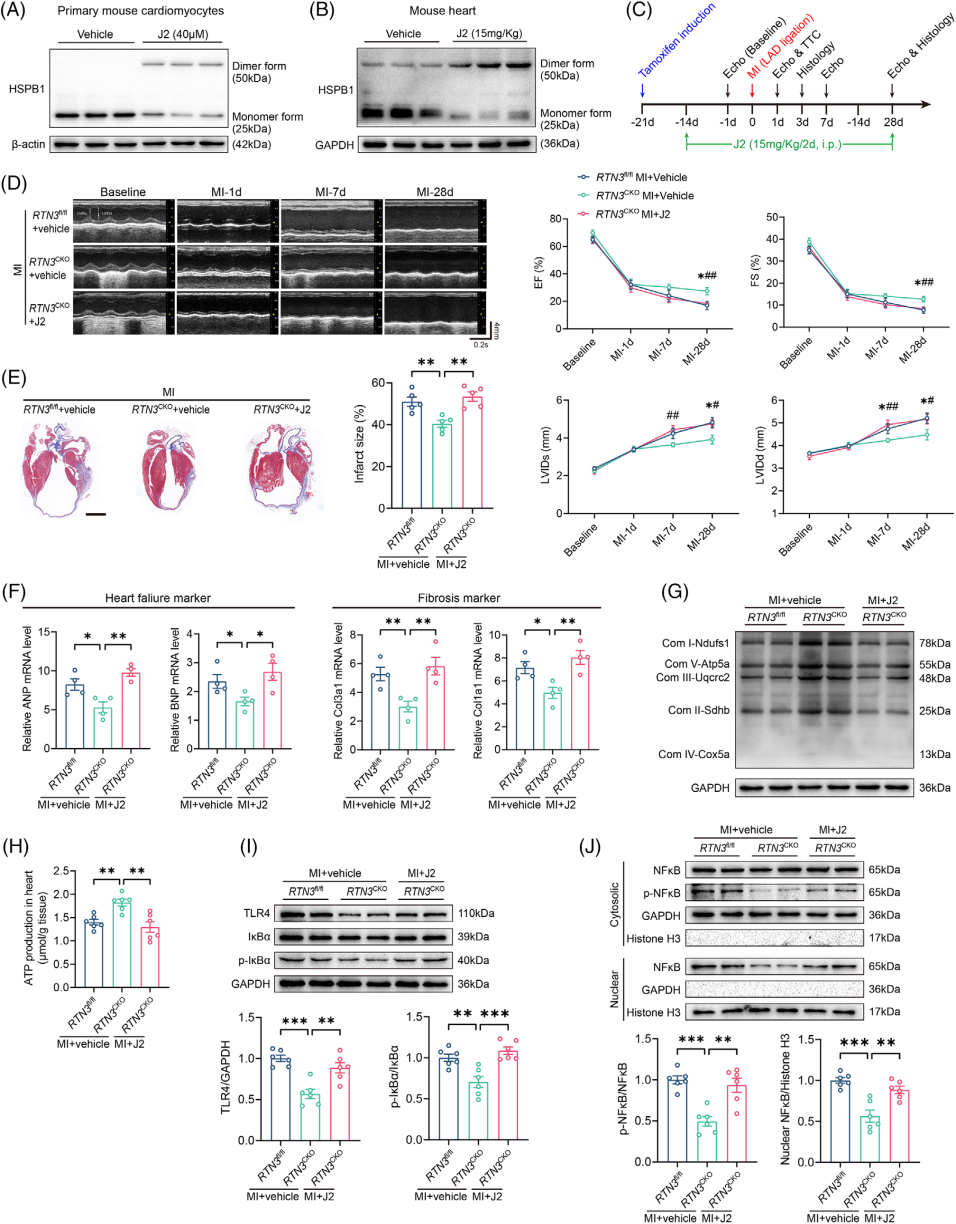
Heat shock protein beta‐1 (HSPB1) inhibition abolishes the ameliorative effect of reticulon 3 (*RTN3*) knockout on heart failure (HF) after myocardial infarction (MI). (A) Representative western blots of HSPB1 different forms in primary mouse cardiomyocytes treated with J2. (B) Representative western blots of HSPB1 different forms in hearts of mice intraperitoneally injected with J2 for 14 days. (C) The timeline of the experimental design for J2 in vivo application. (D) Representative echocardiography images at the indicated time points before and after J2 in vivo application. Echocardiography parameters, including ejection fraction (EF), fractional shortening (FS), left ventricular internal dimension at systolic phase (LVIDs), and left ventricular internal dimension at diastolic phase (LVIDd), were calculated (*n* = 8 per group). ^*^ *p *< 0.05, *RTN3* ^CKO^ + MI + vehicle versus *RTN3* ^fl/fl^ + MI + vehicle; ^#^ *p *< 0.05, *RTN3* ^CKO^ + MI + J2 versus *RTN3* ^CKO^ + MI + vehicle; ^##^ *p *< 0.01, *RTN3* ^CKO^ + MI + J2 versus *RTN3* ^CKO^ + MI + vehicle. (E) Representative Masson trichrome staining images and quantification of infarct size 28 days post‐MI (*n* = 5 per group). Scale bar = 2 mm. (F) Relative mRNA levels of ANP, BNP, Col1a1, and Col3a1 in hearts 28 days post‐MI (*n* = 4 per group). (G) Representative western blots of mitochondrial electron transport chain (ETC) complex subunits in indicated groups. (H) ATP production in the hearts of indicated mice (*n* = 6 per group). (I) Representative western blots and quantitative analysis of TLR4 and p‐IκBα in indicated groups (*n* = 6 per group). (J) Representative western blots and quantitative analysis of p‐NFκB or NFκB in cytoplasm and nucleus (*n* = 6 per group). Data are presented as mean ± standard error of the mean (SEM). Statistical significance was assessed by one‐way analysis of variance (ANOVA) with a Bonferroni post hoc test. ^*^ *p *< 0.05, ^**^ *p *< 0.01, ^***^ *p *< 0.001.

Owing to the mediating role of HSPB1 in RTN3‐driven cardiac dysfunction, we determined whether pharmacological inhibition of HSPB1 could counteract the protective effects of *RTN3* knockout on HF after MI. First, in HL‐1 cells, with the increase of J2 concentration, the abnormal dimer form of HSPB1 increased and the monomer form decreased gradually (Figure S7A). When J2 concentration reached 40 μM, the cell viability decreased significantly (Figure S7B). Consistent with the results of directly knocking down monomer HSPB1 using siRNA shown in Figure S6D, J2 treatment increased TLR4 expression (Figure S7C). Similarly, J2 treatment reduced the monomeric form of HSPB1, a result that was also validated experimentally in primary mouse cardiomyocytes and wild‐type mice (Figure 6A,B). Furthermore, after 28 days of J2 injection, no significant changes in myocardial collagen content and cardiac ejection function were observed in wild‐type mice except for an increase in LV diameter during diastole (Figure S7D,E). The in vivo experimental design is shown in Figure 6C. Compared with the *RTN3* ^CKO^ + MI + vehicle group, J2 significantly inhibited the protective effect of *RTN3* knockout on systolic function at 28 days post‐MI, which was manifested via reduced EF and FS and elevated LVIDs and LVIDd (Figure 6D). Masson staining demonstrated that *RTN3* knockout reduced the infarct size, whereas J2 significantly blocked this effect (Figure 6E). The aggravated cardiac dysfunction and myocardial fibrosis were further supported by increased mRNA levels of ANP, BNP, Col1a1, and Col3a1 in *RTN3* ^CKO^ mice treated with J2 (Figure 6F).

To further explicit whether HSPB1 inhibition in *RTN3* knockout mice could restrain mitochondrial bioenergetics, we assessed the function and morphology of the mitochondria. Compared with vehicle administration, J2 treatment significantly downregulated the protein levels of mitochondrial ETC complex subunits and ATP production in *RTN3* knockout mice after MI (Figure 6G,H). In line with mitochondrial function, TEM analysis demonstrated that J2 treatment significantly exacerbated the abnormal morphology of myocardial mitochondria in *RTN3* knockout mice, as evidenced by further destruction of mitochondrial cristae (Figure S8A). Regarding inflammatory response after MI, TLR4, p‐IκBα, and NFκB nuclear translocation were increased, and immune cell infiltration were aggravated in J2‐treated *RTN3* ^CKO^ mice compared with *RTN3* ^CKO^ + MI + vehicle mice (Figures 6I,J and S8B).

### 2.7 RBM3 positively regulates RTN3 protein expression by binding to and stabilizing its mRNA

Previous studies reported that in HEK293 cells, RBM3 could bind to *RTN3* mRNA and increase its translation through transacting effects on initiation.^24^ To determine the main reason for the RTN3 elevation in hypoxia‐induced cardiomyocytes, we performed the RNA Co‐IP assay (Figure S9A,B). In H9c2 cells, compared with the immunoglobulin G (IgG) group, RBM3 protein could bind to *RTN3* mRNA; the mRNA content was approximately 10‐fold higher than that in the IgG group (Figure S9C). Subsequent in vitro experiments revealed that upregulation and downregulation of RBM3 significantly increased and decreased RTN3 protein expression, respectively (Figure S9D,E). To further clarify whether RBM3 regulates RTN3 at the protein or mRNA level, we performed the RNA stability assay and found that *RTN3* mRNA stability was decreased in siRbm3‐transfected H9c2 cells compared with control cells (Figure S9F). Moreover, hypoxia increased RBM3 protein expression in NRCMs, which was consistent with the changes in RTN3 (Figure S9G). Together, these data explain, at least in part, that RBM3 is an upstream regulator of RTN3 and positively regulates its protein expression by stabilizing *RTN3* mRNA.

### 2.8 Elevated plasma RTN3 level is associated with decreased cardiac function in patients with acute MI

**FIGURE 7:**
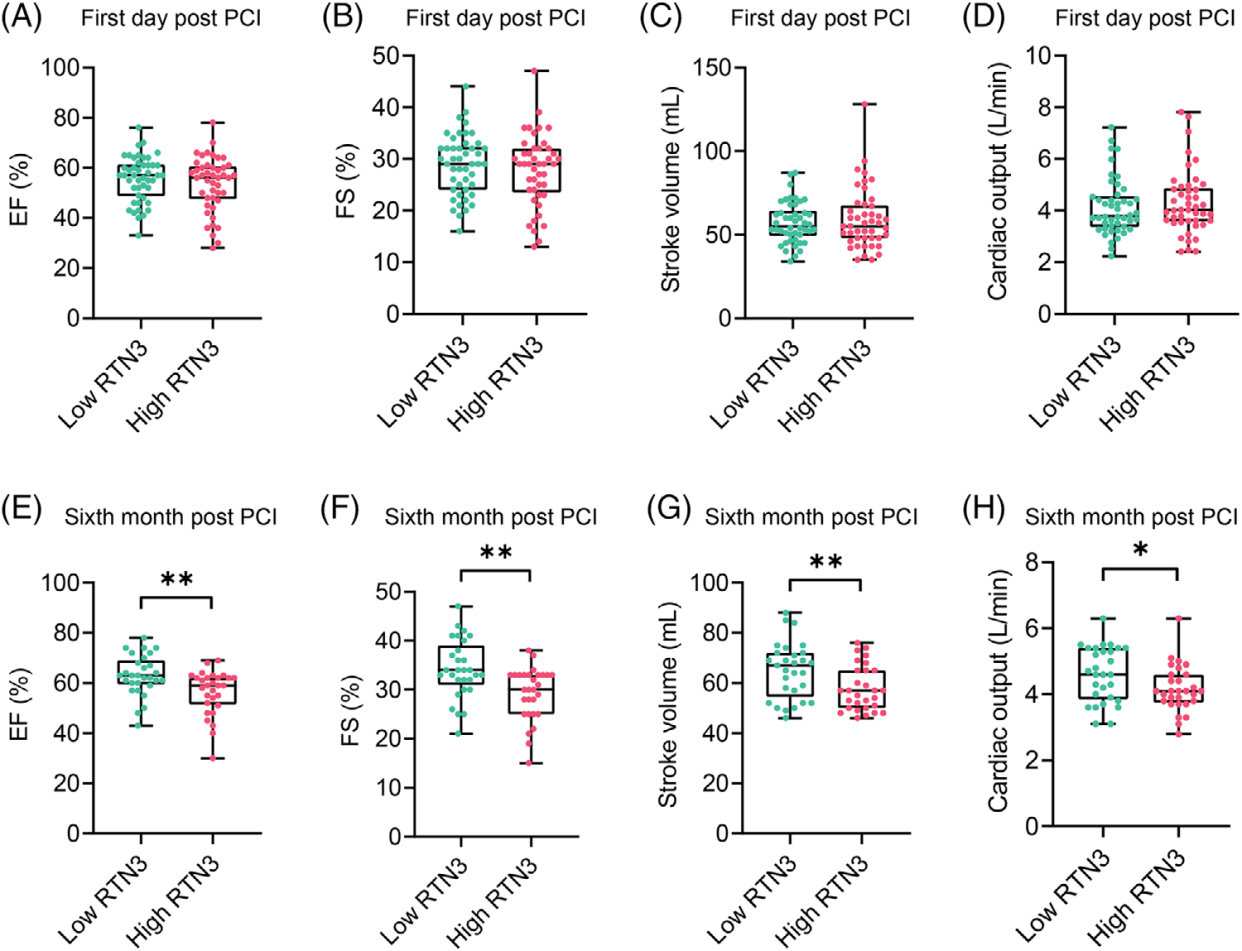
Elevated plasma reticulon 3 (RTN3) level is associated with decreased cardiac function in patients with acute myocardial infarction (AMI). (A–D) Echocardiographic data of patients with AMI in the low RTN3 (*n* = 46) and high RTN3 groups (*n* = 45) at 1 day after percutaneous coronary intervention (PCI). (E–H) Echocardiographic data of patients with AMI in the low RTN3 and high RTN3 groups at 6 months after PCI (*n* = 29 per group). Data are presented as mean ± standard error of the mean (SEM). Statistical significance was assessed by unpaired Student's *t* test. ^*^ *p *< 0.05, ^**^ *p *< 0.01.

To assess the clinical relevance of our findings in mouse MI model, we collected blood samples and clinical data from acute myocardial infarction (AMI) patients undergoing PCI. Because myocardial repair after PCI is a slow process in patients with AMI, this experiment was divided into two parts to explore the relationship between plasma RTN3 levels and cardiac function, including the acute phase of MI (1 day after PCI) and the repair phase of MI (6 months after PCI). There were 91 patients in the acute phase of MI; the detailed characteristics of patients are provided in Table S1. The median plasma RTN3 level was used as the cut‐off point. One day after PCI, patients with AMI were divided into the RTN3 low and high expression groups. The cardiac function parameters EF, FS, stroke volume, and cardiac output were not statistically different between the two groups (Figure 7A–D). Simultaneously, 58 patients were enrolled in the repair phase of MI; the detailed characteristics of patients are listed in Table S2. Interestingly, 6 months after PCI, compared with patients in the RTN3 low expression group, patients in the RTN3 high expression group had significantly decreased cardiac function, as evidenced by reduced EF, FS, stroke volume, and cardiac output (Figure 7E–H). Therefore, these data suggest plasma RTN3 levels at 6 months after PCI in patients with AMI as a potential prognostic marker.

## 3 DISCUSSION

HF after MI is a global health issue with a high mortality rate. The therapeutic effect is limited in clinical settings, and most methods mainly aim to relieve symptoms. Therefore, it is essential to identify potential therapeutic targets to effectively alleviate cardiac dysfunction after MI. The key findings of the present study are as follows: (1) RTN3 expression is increased in the myocardium of patients with HF and mice with MI; (2) RTN3 overexpression leads to decreased cardiac function under physiological conditions and aggravates cardiac dysfunction after MI; (3) cardiomyocyte‐specific *RTN3* knockout alleviates post‐MI HF; (4) mechanistically, *RTN3* deficiency alleviates mitochondrial dysfunction and inflammatory response after MI by upregulating PGC‐1α expression and reducing NFκB nuclear translocation, respectively; (5) HSPB1 inhibition abolishes the ameliorative effect of *RTN3* knockout on HF after MI; (6) elevated plasma RTN3 level is associated with decreased cardiac function in patients with AMI undergo PCI. These findings suggest that RTN3 is a critical therapeutic target for HF after MI.

RTN3, a member of the reticulons family, contains a conserved RHD that plays crucial roles in subcellular localization and protein interaction.^16^, ^18^ Multiple isoforms of mammalian RTN3 are present that can be translated into multiple proteins with different molecular functions.^25^, ^26^, ^27^ Nevertheless, the shorter RTN3, comprising 236 amino acids, is the predominant form of RTN3 in humans. RTN3 exerts pleiotropic roles in amyloid deposition, tumor invasion, and lipid accumulation by interacting with various proteins to alter their enzyme activity, subcellular localization, and protein complex stability.^28^, ^29^, ^30^ However, the mechanism by which RTN3 affects the development of HF after MI remains unexplored. To the best of our knowledge, this is the first study on the role of RTN3 as a regulator of mitochondrial metabolism and inflammation in MI progression.

Given that *RTN3* knockout can obviously alleviate cardiac dysfunction after MI, we aimed to explore the underlying signaling pathways. Transcriptome sequencing and subsequent experiments confirmed that *RTN3* knockout increased the level of mitochondrial ETC complex subunits and improved mitochondrial respiration in the myocardium after MI. However, the specific regulatory mechanisms between RTN3 and mitochondrial function are still worth investigating. Recently, a study reported that RTN3 interacts with and activates CHK2 in a Ca^2+^‐dependent manner, mediates p53 phosphorylation at Ser392, and increases its subsequent nuclear localization, thereby inhibiting the proliferation of hepatocellular carcinoma cells.^29^ Another study on obesity revealed that RTN3 binds to HSPA5 (also called GRP78), which regulates SREBP‐1c activation and promotes lipid synthesis.^30^ These two studies aroused our attention. p53 can promote mitochondrial dysfunction by inhibiting Parkin‐mediated mitophagy,^31^, ^32^ and GRP78, a key ER stress protein, plays an essential role in MI pathogenesis.^33^ Unfortunately, we did not observe any interaction between RTN3 with CHK2 or GRP78 in primary cardiomyocytes (Figure S10).

To further explore the underlying mechanism by which *RTN3* knockout improves cardiac dysfunction in mice, the Co‐IP assay and mass spectrometry were performed to screen RTN3 downstream interacting proteins. HSPB1, a member of the heat shock protein family, is not only responsible for the clearance of misfolded proteins, but also regulates oxidative stress, apoptosis, and inflammatory responses by participating in various signal transduction pathways.^34^, ^35^, ^36^ By constructing truncated plasmids of RTN3 and HSPB1 and extracting the ER proteins, we confirmed that RTN3 could bind to HSPB1 and recruit it to the ER membrane, thereby reducing HSPB1 in the cytoplasm. A previous study reported that cardiomyocyte‐specific *HSPB1* knockout aggravated MI‐induced inflammation by activating the TLR4/NFκB pathway, providing crucial insights into the role of HSPB1.^23^ Subsequent experiments also demonstrated that *RTN3* knockdown alleviated the MI‐induced inflammatory responses via the HSPB1/TLR4/NFκB axis. In addition, analysis of mouse and human RNA‐seq data suggested that the effects of RTN3 on cardiac function were related to PGC‐1α, a key protein involved in mitochondrial biogenesis. TLR4 regulates PGC‐1α after myocardial ischemia, which has been verified in a recent study.^22^ As expected, we also confirmed that *RTN3* knockdown alleviates mitochondrial dysfunction after MI via the HSPB1/TLR4/PGC‐1α axis.

When myocardial ischemia occurs, oxidative phosphorylation in the mitochondrial ETC decreases progressively with time.^37^, ^38^ In particular, the mitochondrial complex I, the initiation complex of the ETC, plays a crucial role in mitochondrial respiration.^22^, ^39^ In our previous study, we found that the upregulation of mitochondrial complex I activity during MI could significantly alleviate mitochondrial dysfunction and reduce reactive oxygen species (ROS) production, thereby improving cardiac function.^40^ Several animal and clinical experiments have demonstrated that PGC‐1α expression is decreased in the myocardium during HF.^41^, ^42^, ^43^ The downregulation of PGC‐1α caused by a pathological injury, such as MI, could lead to the inhibition of mitochondrial gene expression, accompanied by evident mitochondrial structural disorders, abnormal mitochondrial complex activity, and reduced ATP production, further aggravating cardiac dysfunction.^43^, ^44^ Increasing PGC‐1α expression has been proven effective in treating and alleviating various myocardial injuries.^45^, ^46^ Our experiments revealed that RTN3 negatively regulates PGC‐1α expression via the HSPB1/TLR4 pathway. Furthermore, an HSPB1 inhibitor significantly abolished the effect of *RTN3* knockout on PGC‐1α upregulation, thereby hampering mitochondrial biogenesis and aggravating cardiac dysfunction. Our results provide the first evidence that the RTN3/HSPB1/TLR4/PGC‐1α pathway plays an important role in the progression of HF after MI.

MI triggers a robust inflammatory response in the myocardium, and NFκB is a core molecule involved in the inflammatory signaling cascade.^47^, ^48^ NFκB activation and its subsequent nuclear translocation promote the expression of several inflammatory genes.^49^ These inflammatory mediators released by the cardiomyocytes and cardiac resident immune cells could further amplify the inflammatory response.^50^, ^51^ It is important to control inflammation appropriately and promptly to improve myocardial repair after myocardial injury. In the present study, we found that RTN3 reduced cytosolic HSPB1 content by recruiting cytosolic HSPB1 to the ER. The decrease in cytosolic HSPB1 activated the TLR4/NFκB pathway, and the nuclear translocation of NFκB resulted in the expression of chemokines and cytokines, which recruited immune cells to the damaged heart tissue. A variety of HSPs interact with TLRs (especially TLR4) under a wide range of physiological and pathological conditions in vivo to play pro‐inflammatory or anti‐inflammatory roles.^52^ HSPB1 may interact with TLR4 to inhibit the activation of TLR4, thereby achieving anti‐inflammatory effects.^23^, ^53^ In addition, the application of an HSPB1 inhibitor eliminated the protective effect of *RTN3* knockout on inflammation after MI, which further explained the mediating role of HSPB1 in the effect of RTN3 on cardiac function.

Notably, we demonstrated that RTN3 not only plays a crucial physiological role in the development of HF after MI, but also functions as a potential prognostic biomarker. The cohort of patients with MI after PCI was relatively small; however, these patients were very well consistent at baseline. After the occurrence of MI, RTN3 levels in both the plasma and PBMCs were significantly increased; in particular, the elevated plasma RTN3 level was negatively correlated with long‐term cardiac function in patients with AMI undergoing PCI. The association between plasma RTN3 levels and poor patient outcomes suggests the use of RTN3 as a prognostic biomarker in clinical settings. Although RTN3 are mainly localized in the ER, a small portion of RTN3 is also localized together with the Golgi apparatus and plasma membrane. This pleiotropic localization suggests an important involvement of RTN3 in vesicular transport.^54^ Therefore, plasma RTN3 in the normal population may originate from extracellular vesicles produced by various cells, especially vascular endothelial cells. When MI occurs, cardiomyocytes rupture and some ER components are absorbed into the blood, which may cause an increase in plasma RTN3 and be related to patient prognosis. A previous study found that RTN3 is involved in the recruitment and differentiation of monocytes in the process of atherosclerosis.^55^ PBMCs may be subject to the action of chemokines leading to elevated RTN3 expression to recruit to myocardial injury areas and participate in inflammatory responses. Further research is needed on the role and mechanism of RTN3 changes in plasma and PBMCs.

The present study has several limitations. First, the dynamic process by which RTN3 binds and recruits HSPB1 to the ER is still not specific and cannot be evidently observed using current technical methods. Second, consistent with the findings of previous studies, although we verified the response pathway after MI, the specific mechanism of HSPB1 as a negative regulator of TLR4 remains unelucidated. Third, although there are significant differences in clinical data, the cohort has limited clinical data and needs to be confirmed by the other clinical studies in the future. Despite these limitations, our study offers new insights into the role and molecular mechanisms of RTN3 after MI.

In conclusion, the present study provides new evidence that RTN3 is involved in the development and progression of HF after MI. The effects of RTN3 in cardiomyocytes are mediated, at least in part, via HSPB1. Mitochondrial dysfunction and inflammation are important pathogenesis in several cardiovascular diseases, including MI and atherosclerosis. Therefore, targeting RTN3 using AAVs or small molecule inhibitors may be a potential strategy for preventing and treating post‐MI HF and other cardiovascular diseases.

## 4 MATERIALS AND METHODS

An expanded methods section is available in the Supporting Information.

### 4.1 Animals

C57BL/6J mice were purchased from the Experimental Animal Center of Air Force Medical University. All mice were housed in a specific pathogen‐free environment under a 12 h light/dark cycle with ad libitum access to water and food. Mice with the same genotype were randomly grouped. Blinding was performed for all in vivo experiments.

### 4.2 AAV construction and transfection

AAV9 encoding mouse *RTN3* (AAV9‐*RTN3*), controlled by cTnT, was used to upregulate RTN3 expression in mouse hearts. AAV9‐*RTN3* and negative control AAV9‐Ctrl were constructed by GeneChem Technology. Mice randomly received an intrathoracic injection of either AAV9‐*RTN3* or AAV9‐Ctrl at a dose of 2 × 10^11^ v.g./mouse. Three weeks after the injection, heart samples were collected to measure mRNA and protein expression.

### 4.3 Generation of cardiomyocyte‐specific *RTN3* knockout mice

*RTN3* ^fl/fl^ mice were constructed by Shanghai Model Organisms Center, Inc. using the CRISPR‐Cas9 method. In brief, for the mouse *RTN3* transcript (NM_053076.3), Cas9 mRNA and sgRNA were obtained via in vitro transcription. The in‐fusion cloning method was used to construct the donor vector. Then, Cas9 mRNA, gRNA, and donor vector were microinjected into the zygotes of C57BL/6J mice to obtain chimeric mice. To specifically knockout *RTN3* in the cardiomyocytes, the chimeric mice were mated with *αMyh6‐Cre* transgenic mice (Jackson Laboratory). The primer sequences for mouse genotyping were as follows: forward, 5′‐GAGAATGGCTTAGAATGGTGAAAA‐3′; and reverse, 5′‐GAGAGGGGGAATAGAAGTTAGAGA‐3′, which yielded 399 bp products for *RTN3* ^fl/fl^, 335 bp products for the *RTN3* wild‐type and both 399 and 335 bp products for *RTN3* ^fl/+^; forward, 5′‐GGTAGAAATGGCCAAGACTCAGAC‐3′; and reverse, 5′‐CCCCCTCCCGACAAAAAGATA‐3′, which yielded 300 bp products for *αMyh6‐Cre*.

To induce Cre expression in *RTN3* ^CKO^ mice, tamoxifen (30 mg/kg, Sigma–Aldrich) dissolved in corn oil was intraperitoneally injected daily for 5 consecutive days. *RTN3* ^fl/fl^ mice were used as control mice and were exposed to the tamoxifen injection. Mice were normally fed for 2 weeks to allow tamoxifen clearance, followed by measurement of RTN3 protein and mRNA levels.

### 4.4 Mouse MI model

MI was modeled surgically in mice aged 8–10 weeks by ligating the coronary artery LAD branch, as previously described.^56^ Briefly, mice were anesthetized with 2% isoflurane, and the heart was quickly squeezed through the chest incision to avoid pneumothorax. The LAD of the coronary artery was ligated 2–3 mm below the origin using a 6‐0 silk suture. Mice in the sham group underwent the same operation but without ligation. Changes in the electrocardiogram were an important indicator of successful induction of MI in mice.

### 4.5 Statistical analysis

Continuous variables are presented as mean ± standard error of the mean, and categorical variables are presented as numbers and percentages. All data were analyzed using GraphPad Prism 8.0 (GraphPad Software). For continuous variables, an unpaired two‐tailed Student's *t* test was used to analyze the differences between the two groups, and one‐way analysis of variance was performed with a Bonferroni post hoc test for multiple group comparisons. For categorical variables, the chi‐square test was performed when the total number was >40, and the Fisher's exact test was performed when the total number was <40. A *p‐*value < 0.05 was considered statistically significant.

## AUTHOR CONTRIBUTIONS

Y.L. and M.Z. conceived and designed the study. B.Q., T.L., and H.L. performed most experiments and analyzed data. L.H., R.F., and D.W. participated in animal experiments. T.P., G.R., and D.G. participated in cellular experiments. M.L. and Q.W. participated in clinical data collection. B.Q. wrote the original draft of the manuscript. Y.L. and M.Z. revised the manuscript. All of the authors have read and approved the final manuscript.

## CONFLICT OF INTEREST STATEMENT

The authors declare they have no conflicts of interest.

## ETHICS STATEMENT

All animal experiments were approved by the Animal Care and Use of Air Force Medical University (approval number: IACUC‐20211199) and were conducted in accordance with t the Guide for the Care and Use of Laboratory Animals issued by the US National Institutes of Health (NIH Publications, 8th edition, 2011). Human studies were conducted according to the principles of the Declaration of Helsinki and were centrally approved by the Ethics Committee of Tangdu Hospital, Air Force Medical University (approval number: K202106‐09). Written informed consent was obtained from all participants.

## Supporting information

Supporting Information

## Data Availability

The data underlying this article will be shared on reasonable request to the corresponding author.
