# Supplementary material for: Reticulon 3 deficiency ameliorates post‐myocardial infarction heart failure by alleviating mitochondrial dysfunction and inflammation
Source: MedComm (2020). 2024 Feb 28;5(3):e503. doi: 10.1002/mco2.503 (PMC10901281; doi:10.1002/mco2.503)
Supplement: Supplementary file 1 — Supporting Information [file MCO2-5-e503-s001.pdf]

Supplementary Information

Reticulon 3 deficiency ameliorates post-myocardial infarction heart failure by alleviating mitochondrial dysfunction and inflammation

Short title: Targeting RTN3 for myocardial infarction.

Bingchao Qi[1,#], Tiantian Li[1,#], Haixia Luo[1,#], Lang Hu[1], Renqian Feng[1], Di Wang[1], Tingwei Peng[1], Gaotong Ren[1], Dong Guo[1], Mingchuan Liu[1], Qiuhe Wang[1], Mingming Zhang[1,*], and Yan Li[1,*]

[1] Department of Cardiology, Tangdu Hospital, Air Force Medical University, Xi'an, 710038, People's Republic of China

[#] These authors contributed equally

[*] **Correspondence**

Yan Li, Department of Cardiology, Tangdu Hospital, Air Force Medical University, Xi'an, Shaanxi, 710038, China. Tel: 86-29-84717648. E-mail: profleeyan@163.com.

Mingming Zhang, Department of Cardiology, Tangdu Hospital, Air Force Medical University, Xi'an, Shaanxi, 710038, China. E-mail: winterzhang3@163.com.

Funding information

National Natural Science Foundation of China, Grant/Award Numbers: 82070385; Shaanxi Province Science and Technology New Star Project, Grant/Award Numbers: 2022KJXX-107; The Innovation Project of Tangdu Hospital, Grant/Award Numbers: 2019GJHZ002.

Supplemental Methods

Human samples

Human blood samples were collected from subjects without MI, patients with MI before PCI, and patients with MI 1 day and 6 months after PCI. The detailed characteristics of patients are provided in Table S1, Table S2 and Table S3. Further, heart samples are obtained from patients who underwent coronary bypass surgery.

Blood samples were collected using EDTA-coated tubes and centrifuged at 3000 g for 10 min. The upper fluid (plasma), was transferred into a new centrifuge tube and stored at -80°C. Plasma RTN3 levels were measured using a Human RTN3 ELISA Kit (FineTest, China), according to the manufacturer's instructions. Mononuclear cells were extracted from the patient's blood using a Human Peripheral Blood Mononuclear Cell Isolation Kit (Solarbio, China), according to the manufacturer's instructions.

Cell culture and hypoxia stimulation

NRCMs and NRCFs were isolated from 1-2-day-old Sprague Dawley rats obtained from the Experiment Animal Center of Air Force Medical University. Heart tissues were cut into pieces, washed twice with PBS to remove blood, and digested with Type I Collagenase solution (1 mg/ml, Gibco, USA) 5-6 times at 37°C. The digestion process was terminated by adding Dulbecco's modified Eagle medium (DMEM) supplemented with 10% fetal bovine serum (FBS, Gibco, USA). Owing to different cell adhesion time, NRCMs and NRCFs were separated after 2 hours of differential adhesion. Then, the two primary cells were cultured for an additional 48 hours and subjected to follow-up experiments. The H9c2 cell line, derived from the embryonic rat heart ventricle, was used for truncated plasmid transfection experiments.

To stimulate hypoxia, the cells were transferred to sugar-free and serum-free DMEM to simulate nutrient deprivation. Then, the cells were cultured in a hermetic incubator (5% CO_2, 95% N_2) at 37°C for 6 hours.

Download of public datasets

RNA-seq data for human heart tissues were downloaded from the GEO database.[1] GSE161472, GSE46224 and GSE116250 were high-throughput sequencing data sets

of myocardial samples from patients with HF. The GSE161472 dataset contained transcriptome differences between patients with HF and controls in four heart chambers. The GSE46224 and GSE116250 datasets included transcriptome data in myocardial samples from patients with ICM and their controls.

Quantitative real-time PCR (qRT-PCR)

Heart tissues or cells were washed twice with ice-cold PBS. Then, total RNA was extracted using RNAiso Plus (TaKaRa, Japan) according to the manufacturer's instructions. cDNA was prepared via reverse transcription using a PrimeScript RT reagent kit (TaKaRa, Japan). RT-PCR was performed using TB Green Premix Ex Taq II (TaKaRa, Japan) on a CFX96 real-time PCR system (Bio-Rad, USA). The thermal cycling conditions were as follows: denaturation at 95°C for 30 s, 40 cycles at 95°C for 5 s and at 60°C for 30 s. The mRNA expression of target genes was normalized to that of β-actin. Data were analyzed using the standard comparative CT method. The primer sequences are shown in Table S4.

Western blot

Total proteins were isolated from heart tissues or cells with RIPA buffer (Beyotime, China) supplemented with a protease inhibitor cocktail (Beyotime, China). Nuclear and cytoplasmic proteins were prepared using a Nuclear and Cytoplasmic Protein Extraction Kit (Beyotime, China). ER proteins were prepared using an Endoplasmic Reticulum Protein Extraction Kit (Solarbio, China). Equal amounts of protein were separated by SDS-PAGE. Then, gels were transferred onto polyvinylidene fluoride membranes and incubated with primary antibodies overnight at 4°C. Membranes were washed and incubated with HRP-conjugated secondary antibodies at room temperature for 1 h. The proteins were detected on a chemiluminescence system (Bio-Rad, USA), and immunoblot intensity was analyzed using LabImage software (Bio-Rad, USA). β-actin or GAPDH was used as the internal control for total protein. Histone-H3 and calreticulin were used as the internal controls for nuclear proteins and ER proteins, respectively. The antibodies used and the dilution ratio are shown in Table S4.

Echocardiography

Cardiac function was detected using the Vevo 2100 echocardiography system

(VisualSonics, Canada). Mouse chest hair was removed prior to the experiment. Then, the mice were anesthetized with isoflurane (2.5% for induction and 1.5% for maintenance) and placed on a warm heated platform. After alignment in the B-mode with the papillary muscles, cardiac function was measured in the M-mode. During the procedure, a real-time ECG detector was used to ensure that the heart rate of each mouse was maintained at 400-450 beats per minute. The cardiac parameters LVIDs, LVIDd, EF, and FS were obtained using Vevo 2100 software (VisualSonics, Canada). Measurements were performed for at least four cardiac cycles per mouse. Echocardiography and subsequent analysis were performed by a researcher who was blinded to the treatments.

Histological analysis

Mouse hearts were washed twice with ice-cold PBS, fixed overnight with 4% paraformaldehyde, embedded in paraffin, and cut into 5 μm-thick sections. Masson trichrome staining was performed on heart sections to assess the cardiac collagen content and infarct size. For mice without MI, cardiac collagen content was expressed as percentage collagen volume of the left ventricle volume. For mice with MI, infarct size was calculated as the total infarct circumference divided by the total left ventricle circumference. In addition, at 24 hours post-MI, mouse hearts were rapidly cut into four consecutive 1.0 mm-thick sections from the apex to the level of the suture and perpendicular to the long axis of the heart. Sections were incubated with 1% TTC staining solution (Sigma-Aldrich, USA) at 37°C for 20 min, and then gently shaken every 5 min to ensure uniform contact between the sections and staining solution. The TTC-stained area (non-infarcted myocardium) and TTC-negative area (infarcted myocardium) were measured using ImageJ software.

Immunofluorescence

For immunofluorescent staining of tissues, sections were permeabilized with 0.2% Triton X-100 for 10 min, blocked with 2% BSA solution for 30 min at room temperature, and incubated overnight with the primary antibody at 4°C. After washed three times with PBS, tissue sections were incubated with fluorescent secondary antibodies for 1 h at room temperature in the dark. DAPI staining solution (Beyotime, China) was used to

stain the nucleus. Immunofluorescence images were obtained with a confocal laser scanning microscope (A1R MP+ Confocal Microscope, Nikon, Japan) and analyzed with ImageJ software. For cell immunofluorescence staining, cardiomyocytes were seeded in confocal dishes at suitable densities. After the cells were treated, they were fixed with pre-cold methanol for 10 min, then washed twice with PBS. Subsequent blocking, primary antibody incubation, secondary antibody incubation, and DAPI staining were consistent with tissue immunofluorescence methods.

Transmission electron microscopy (TEM)

Myocardial mitochondrial cristae morphology was observed with TEM, as described previously.[2] Mouse hearts were washed twice with ice-cold PBS and fixed overnight in 2.5% glutaraldehyde in 0.1 mol/L sodium cacodylate buffer at room temperature. Left ventricles were cut into longitudinal strips and postfixed with 1% osmium tetroxide in 0.1 mol/L sodium cacodylate buffer for 2 h at 4°C. The samples were dehydrated, embedded, cut into ultrathin sections, and stained with uranyl acetate and lead citrate. Mitochondrial ultrastructure images were obtained with a transmission electron microscope (JEM-1230, JEOL, Ltd., Japan).

Plasmid transfection, gene silencing, and adenovirus infection

All plasmids and small interfering RNAs used in this experiment were synthesized by Tsingke Biotechnology (Beijing, China). Adenovirus and its controls were designed and constructed by Hanbio Technology (Shanghai, China). For plasmid transfection, H9c2 myoblasts were transfected with indicated plasmid using Lipofectamine 2000 (Invitrogen, USA), according to the manufacturer's protocol. For gene silencing, cardiomyocytes were transfected with 10 μmol/L siRNA using Lipofectamine RNAiMAX (Invitrogen, USA), following the instructions provided by the supplier. For adenovirus infection, adenovirus and its control were added directly to the DMEM at a multiplicity of infection (MOI) of 50. Six hours after cell transfection or infection, the medium was changed to fresh DMEM supplemented with 10% FBS and continued for 48 h. The sequences of the siRNAs are shown in Table S4.

Co-immunoprecipitation (Co-IP)

Co-IP was performed using a Pierce Classic Magnetic IP/Co-IP Kit (Thermo Fisher

Scientific, USA) according to the manufacturer's protocol. Briefly, NRCMs or H9c2 cells were washed with ice-cold PBS and lysed with IP lysis supplemented with a protease inhibitor cocktail. Cell lysates were incubated with chosen primary antibody for IP overnight at 4ºC. The antigen-antibody complexes were mixed with Protein A/G magnetic beads for 2 hours at room temperature. Beads-antigen-antibody complexes were collected with a magnetic separator and washed twice with IP Wash Buffer and once with purified water. Antigens were eluted with Elution Buffer and denatured by boiling with loading buffer at 95ºC for 10 min. IgG was used as a negative control for subsequent western blot or mass spectrometry analysis.

Mass spectrometry analysis

Liquid chromatography-tandem mass spectrometry (LC-MS/MS) analysis was performed by Novogene Genetics (Beijing, China) as previously described.[2] The protein bands were cut, destained, dehydrated, and digested. The separated peptides were analyzed by Q Exactive HF-X mass spectrometer (Thermo Fisher Scientific, USA). The ion spray voltage was set to 2.3 kV, and the full mass spectrum scan range was set to 350-1500 m/z with primary mass spectrum resolution of 60000 (200 m/z). The top 40 precursor ions in full scan were fragmented using high-energy collision cleavage for secondary mass spectrometry with a secondary mass spectral resolution of 15000 (200 m/z). The raw files obtained by detection are directly imported into the Proteome Discoverer2.2 software for database search, peptide quantification and protein quantification.

RNA sequence

RNA sequence was performed by Novogene Genetics (Beijing, China), as previously described.[3] Total RNA was extracted from *RTN3*^{flfl} and *RTN3*^{CKO} mice, and the integrity of RNA was assessed with an Agilent 2100 bioanalyzer (Agilent Technologies, USA). After the RNA library was constructed, it was quantified by Agilent 2100 bioanalyzer and qPCR method. Then the library was qualified, and high-throughput sequencing was performed with the Illumina NovaSeq 6000 (Illumina, USA). For the mouse genome (GRCm38), transcriptional reads were mapped using a HISAT2 tool. Three biological replicates were set up for each set of samples, and DESeq2 package (1.20.0) was used

to perform differential expression analysis between the two groups. For functional analysis, GO enrichment analysis, KEGG pathways enrichment analysis, Reactome pathways enrichment, and GSEA were implemented by the clusterProfiler R package (3.8.1).

Measurement of mitochondrial respiratory function

The mitochondrial oxygen consumption rate (OCR) was measured with an extracellular flux analyzer (Agilent Seahorse Bioscience, USA). In brief, NRCMs were laid on a 24-well seahorse assay plate (Agilent Seahorse Bioscience, USA). Six hours after adenovirus transfection, the medium was changed to fresh DMEM supplemented with 10% FBS and continued for 48 h. Cells are treated with hypoxia for 6 hours prior to the test. The working concentrations of the four inhibitors were as follows: 1 μmol/L oligomycin, 1 μmol/L FCCP, 0.5 μmol/L rotenone, and 0.5 μmol/L antimycin A. The Wave software (Agilent Seahorse Bioscience, USA) was used for data collection. XF Cell Mito Stress Test Generator software (Agilent Seahorse Bioscience, USA) was used for OCR data analysis.

RNA immunoprecipitation (RIP)

RIP experiments were performed using an RNA-Binding Protein Immunoprecipitation Kit (Merck Millipore, Germany) according to the manufacturer's protocol. Briefly, an adequate amount of H9c2 cells were lysed with RIP lysis buffer supplemented with a protease inhibitor cocktail and RNase inhibitor. The RBM3 antibody was mixed with the magnetic beads and incubated for 1 hour at room temperature to form the antibody-beads complexes. The complexes were washed six times with RIP wash buffer, and mixed with cell lysates overnight at 4ºC to obtain beads-antigen-antibody complexes. The complexes were collected with a magnetic separator and washed six times with RIP wash buffer. Purification of RNA was performed with proteinase K buffer at 55ºC for 30 min. IgG was used as a negative control for subsequent IP verification or qPCR analysis.

mRNA stability evaluation

To assess the stability of mRNA, we treated cells with 5 μmol/L actinomycin D (MedChemexpress, USA). Cells were harvested at indicated time points. RNA was

extracted, reverse transcribed and analyzed by qPCR for its half-life.

References

1. Edgar R., Domrachev M., Lash A.E. (2002). Gene expression omnibus: ncbi gene expression and hybridization array data repository. Nucleic Acids Res. *30*, 207-210.
2. Qi B., He L., Zhao Y., Zhang L., He Y., Li J., Li C., Zhang B., Huang Q., Xing J., et al. (2020) Akap1 deficiency exacerbates diabetic cardiomyopathy in mice by ndufs1-mediated mitochondrial dysfunction and apoptosis. Diabetologia ***63***, 1072-1087.
3. Liu N., Kataoka M., Wang Y., Pu L., Dong X., Fu X., Zhang F., Gao F., Liang T., Pei J., et al. (2021). LncRNA Lnchrt preserves cardiac metabolic homeostasis and heart function by modulating the lkb1-ampk signaling pathway. Basic Res. Cardiol. *116*, 48.

Supplemental Figures

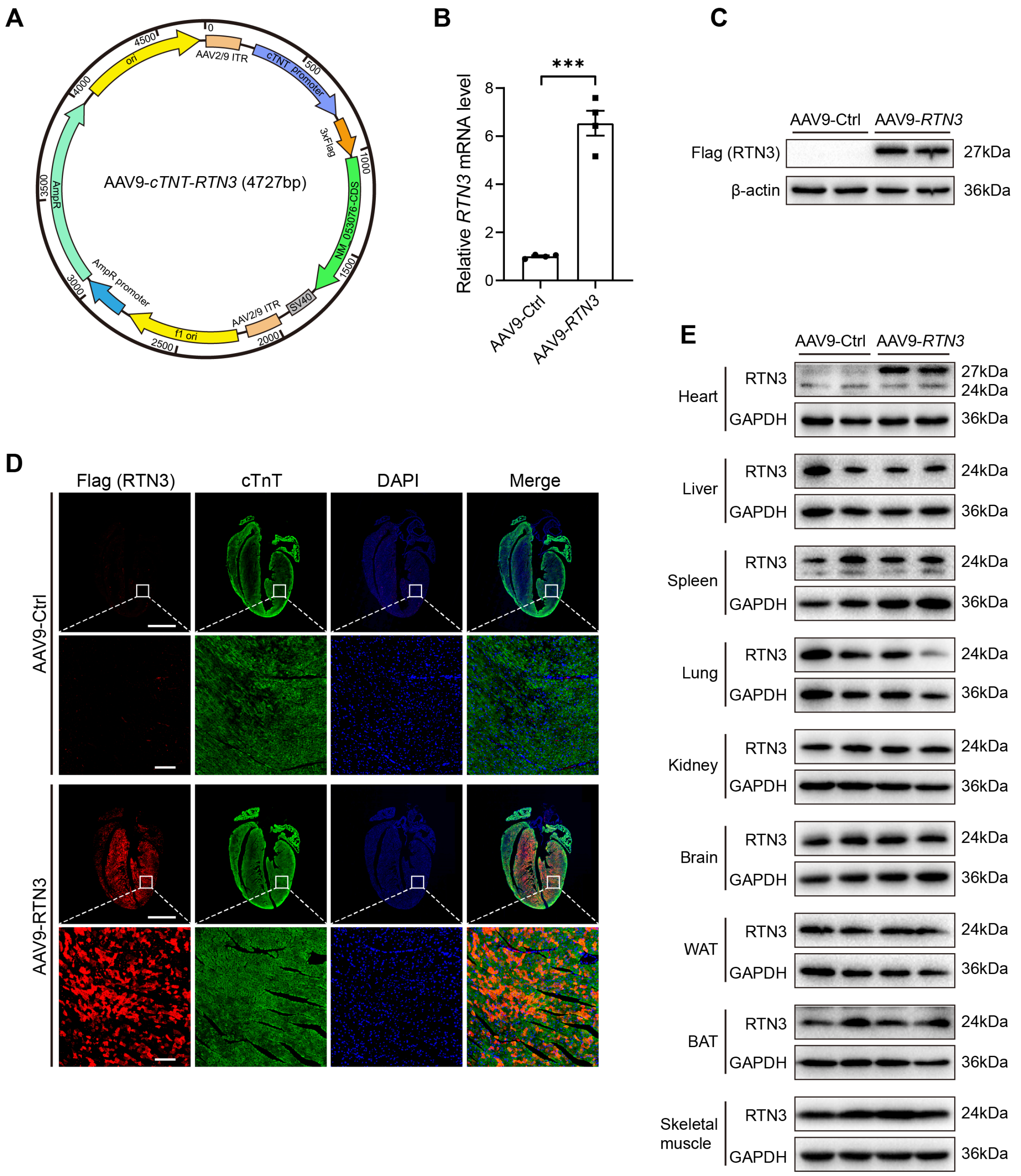

Figure S1. Adeno-associated virus 9 (AAV9) is used to generate cardiomyocyte-specific RTN3 overexpression mice.

(A) The map of the AAV9-*cTnT-RTN3* used in the experiments. (B) Relative mRNA level of *RTN3* in hearts of mice injected with AAV9-*RTN3* or AAV9-Ctrl for 3 weeks (n=4 per group). (C) Representative western blots of Flag (RTN3) in the indicated group. (D) Immunofluorescence images of Flag (RTN3) expression in whole hearts of mice 1 day after MI. cTnT (green) was used as a cardiomyocyte marker, and DAPI (blue) was

used to stain the nuclei. Overall scale bar=2 mm, and local scale bar=100 μm. (E) Representative western blots showing RTN3 protein in different tissues of mice injected with AAV9-*RTN3* or AAV9-Ctrl for 3 weeks. Data are presented as mean ± SEM. Statistical significance was assessed by unpaired Student's *t* test. ***P<0.001.

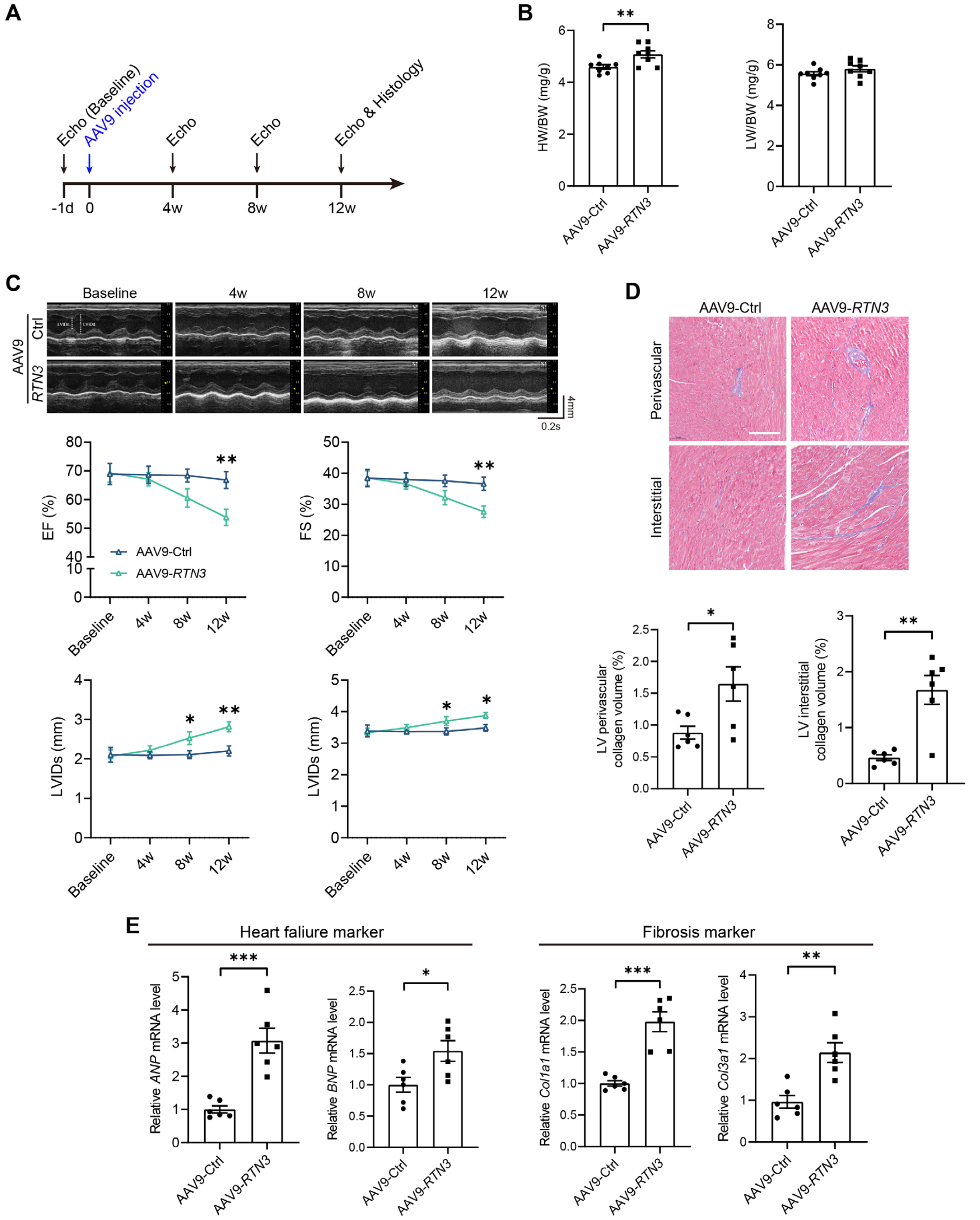

Figure S2. Cardiomyocyte-specific RTN3 overexpression leads to decreased cardiac function in mice under normal physiological conditions.

(A) The timeline of the experimental design for RTN3 overexpression in mice. (B) Heart weight/body weight (HW/BW), and lung weight/body weight (LW/BW) were evaluated 12 weeks after AAV9 injection (n=8 per group). (C) Representative M-mode echocardiography images at the indicated time points. Echocardiography parameters,

including EF, FS, LVIDs, and LVIDd, were calculated (n=10 per group). EF, ejection fraction; FS, fractional shortening; LVIDs, left ventricular internal dimension at systolic phase; LVIDd, left ventricular internal dimension at diastolic phase. (D) Representative Masson trichrome staining images and quantification of collagen volume 12 weeks after AAV9 injection (n=6 per group). Scale bar=100 μm. TTC, triphenyl tetrazolium chloride. (E) Relative mRNA levels of ANP, BNP, Col1a1, and Col3a1 in hearts 12 weeks after AAV9 injection (n=6 per group). Data are presented as mean ± SEM. Statistical significance was assessed by unpaired Student's *t* test. *$P<0.05$, **$P<0.01$, ***$P<0.001$.

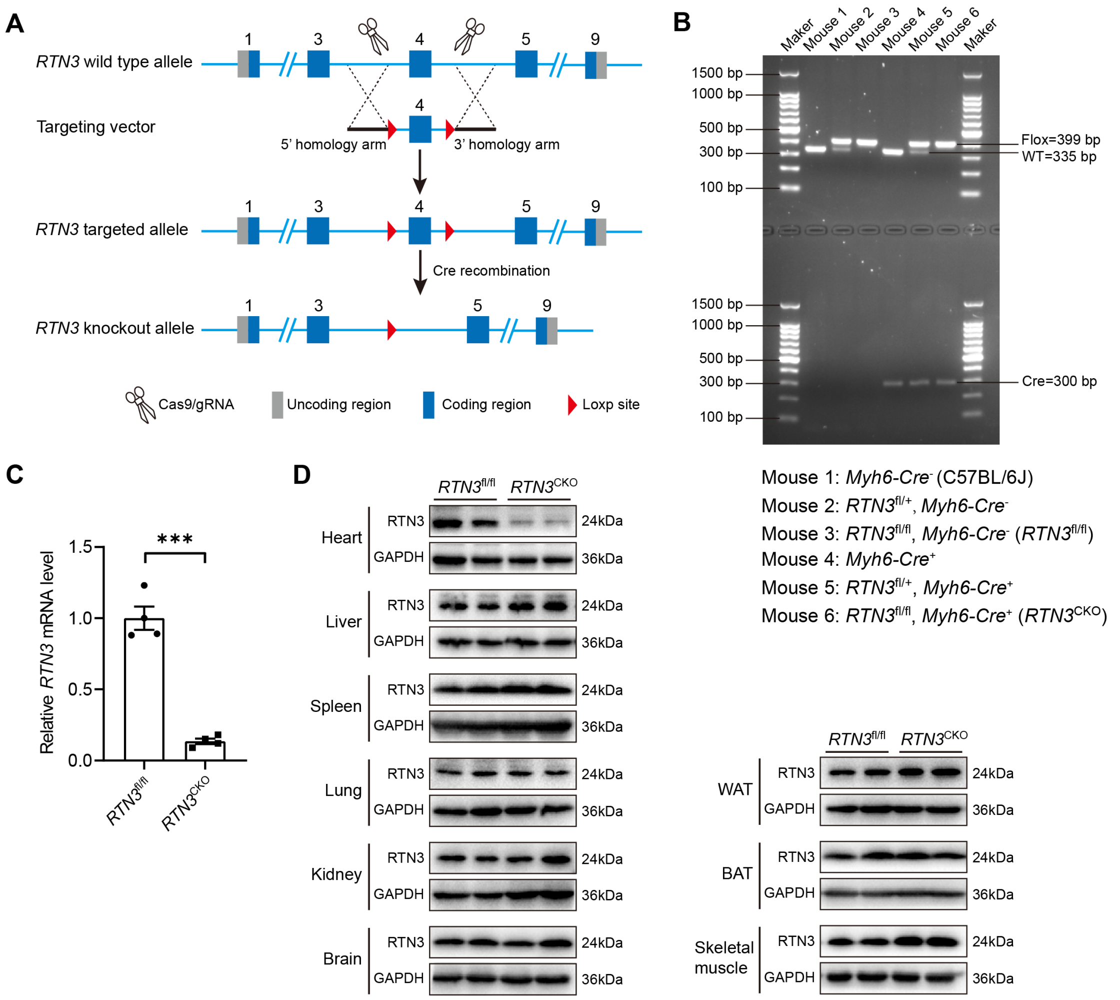

Figure S3. CRISPR/Cas9 method is used to generate cardiomyocyte-specific *RTN3* knockout mice.

(A) Schematic of the strategy to generate *RTN3*$^{\text{fl/fl}}$ and *RTN3*^{CKO} mice. (B) PCR genotyping of the *RTN3*$^{\text{fl/fl}}$ and *RTN3*^{CKO} mice. 335 bp for wild type (WT), 399 bp for *RTN3*$^{fl/fl}$ mice, and 300 bp for *Cre*. (C) Relative mRNA levels of *RTN3* in mouse hearts 3 weeks after tamoxifen injection (n=4 per group). (D) Representative western blots showing RTN3 protein in different tissues of mice injected with tamoxifen for 3 weeks. Data are presented as mean ± SEM. Statistical significance was assessed by unpaired Student's *t* test. ****P*<0.001.

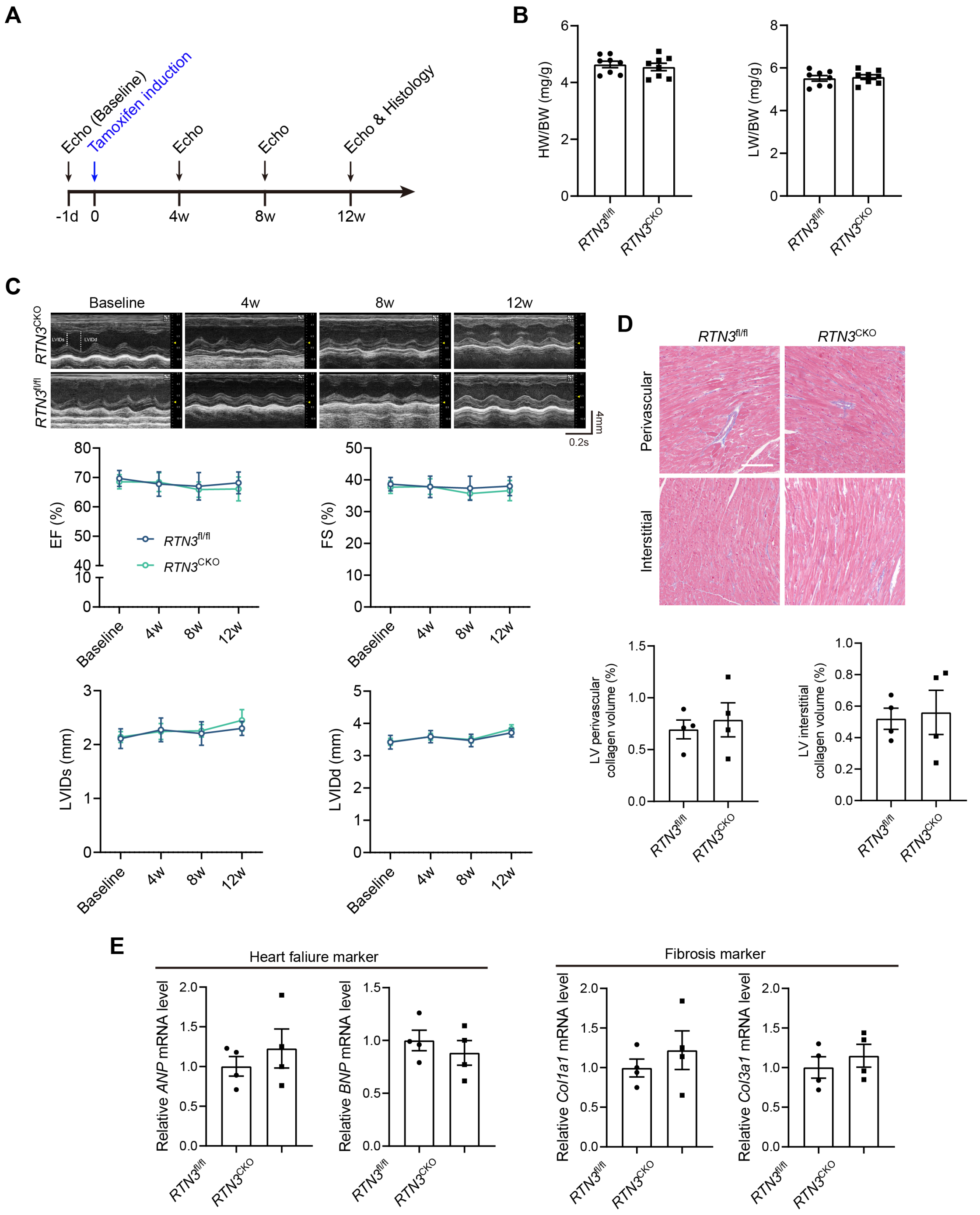

Figure S4. Cardiomyocyte-specific *RTN3* knockout has no effect on cardiac function in mice under normal physiological conditions.

(A) The timeline of the experimental design for *RTN3* knockout in mice. (B) HW/BW and LW/BW were evaluated 12 weeks after tamoxifen injection (n=8 per group). (C) Representative M-mode echocardiography images at the indicated time points. Echocardiography parameters, including EF, FS, LVIDs, and LVIDd, were calculated

(n=8 per group). (D) Representative Masson trichrome staining images and quantification of collagen volume 12 weeks after tamoxifen injection (n=4 per group). Scale bar=100 μm. (E) Relative mRNA levels of ANP, BNP, Col1a1, and Col3a1 in hearts 12 weeks after tamoxifen injection (n=4 per group). Data are presented as mean ± SEM. Statistical significance was assessed by unpaired Student's *t* test.

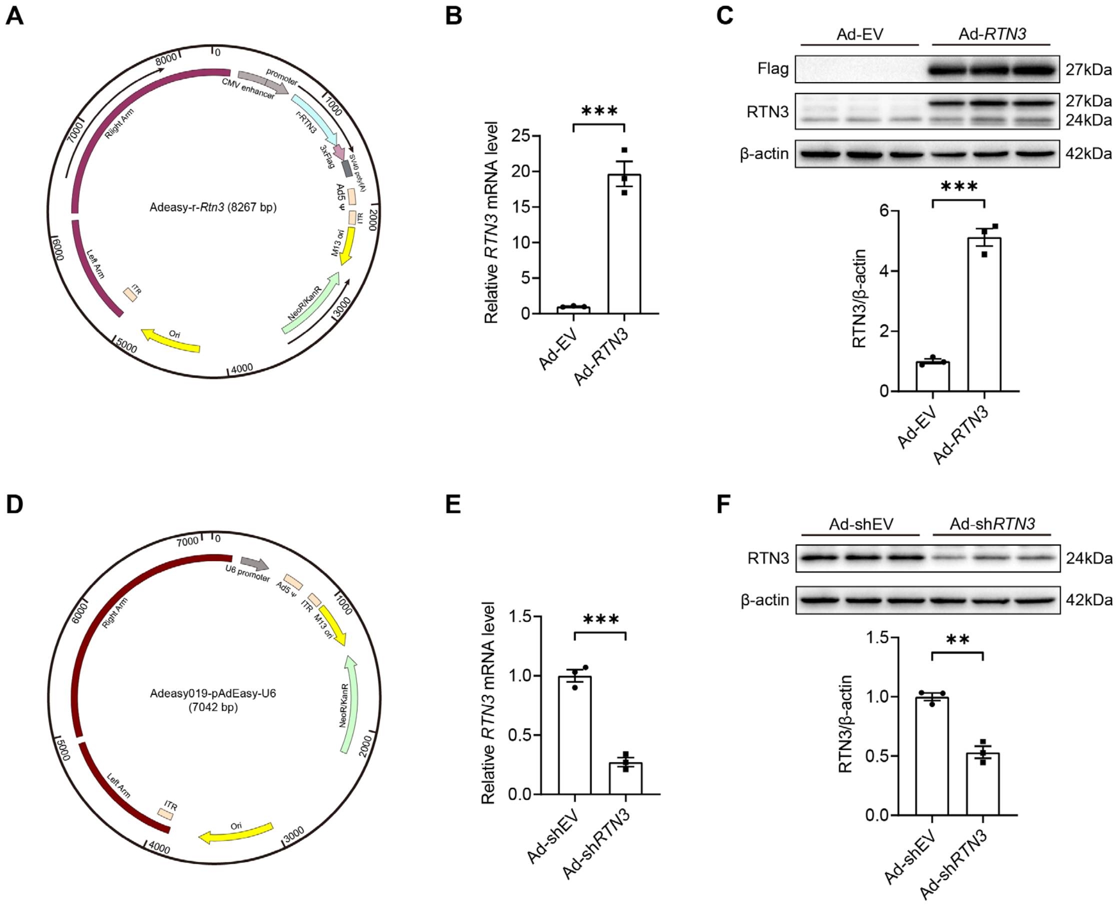

Figure S5. Adenoviruses are used to upregulate or downregulate RTN3 expression in NRCMs.

(A) The map of the Ad-*RTN3* used in the study. (B) Relative mRNA level of *RTN3* in NRCMs transfected with Ad-*RTN3* or empty virus (Ad-EV) for 48 hours (n=3 per group). (C) Representative western blots and quantification of Flag and RTN3 in the indicated group. (D) The map of the Ad-sh*RTN3* used in the study. (E) Relative mRNA level of *RTN3* in NRCMs transfected with Ad-sh*RTN3* or control virus (Ad-shEV) for 48 hours (n=3 per group). (F) Representative western blots and quantification of RTN3 in indicated group. Data are presented as mean ± SEM. Statistical significance was assessed by unpaired Student's *t* test. ***P*<0.01, ****P*<0.001.

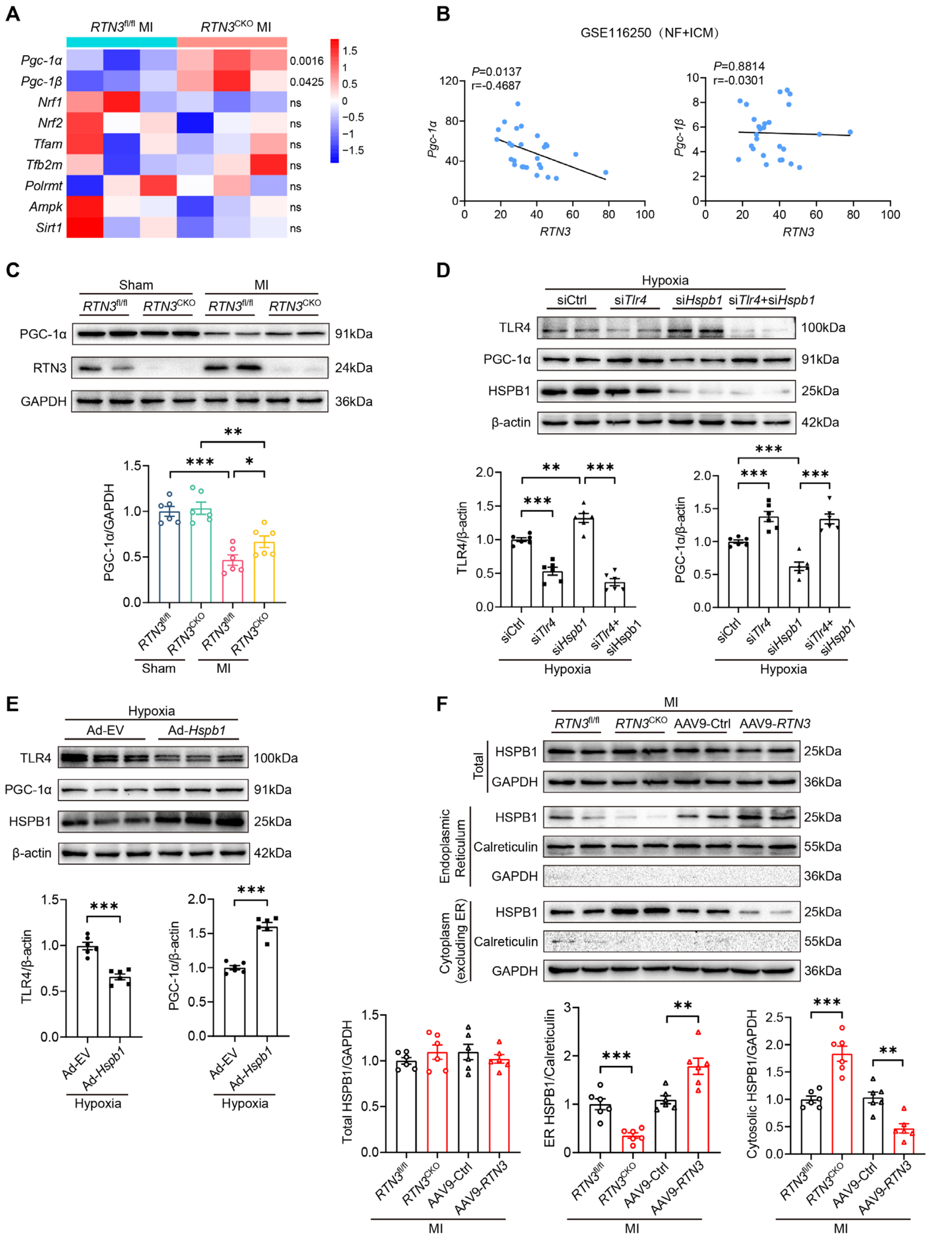

Figure S6. RTN3-mediated HSPB1 translocation regulates post-MI mitochondrial biogenesis via the TLR4/PGC-1α pathway.

(A) Heatmap showing the expression of mitochondrial biogenesis related genes in RNA-seq data. (B) Correlation analysis between *RTN3* and *PGC-1α*/*PGC-1β* expression in human RNA-seq data (GSE116250) using the Pearson correlation coefficient. (C) Representative western blots and quantitative analysis of PGC-1α levels

in indicated mouse hearts (n=6 per group). (D and E) Representative western blots and quantitative analysis of relevant proteins in H9c2 cells transfected with indicated siRNA or plasmid (n=6 per group). (F) Western blot analysis of HSPB1 expression in ER and cytoplasm from the hearts of indicated mice (n=6 per group). Data are presented as mean ± SEM. Data in (C and D) were analyzed by one-way ANOVA with a Bonferroni post hoc test. Others were analyzed by unpaired Student's *t* test. *$P<0.05$, **$P<0.01$, ***$P<0.001$.

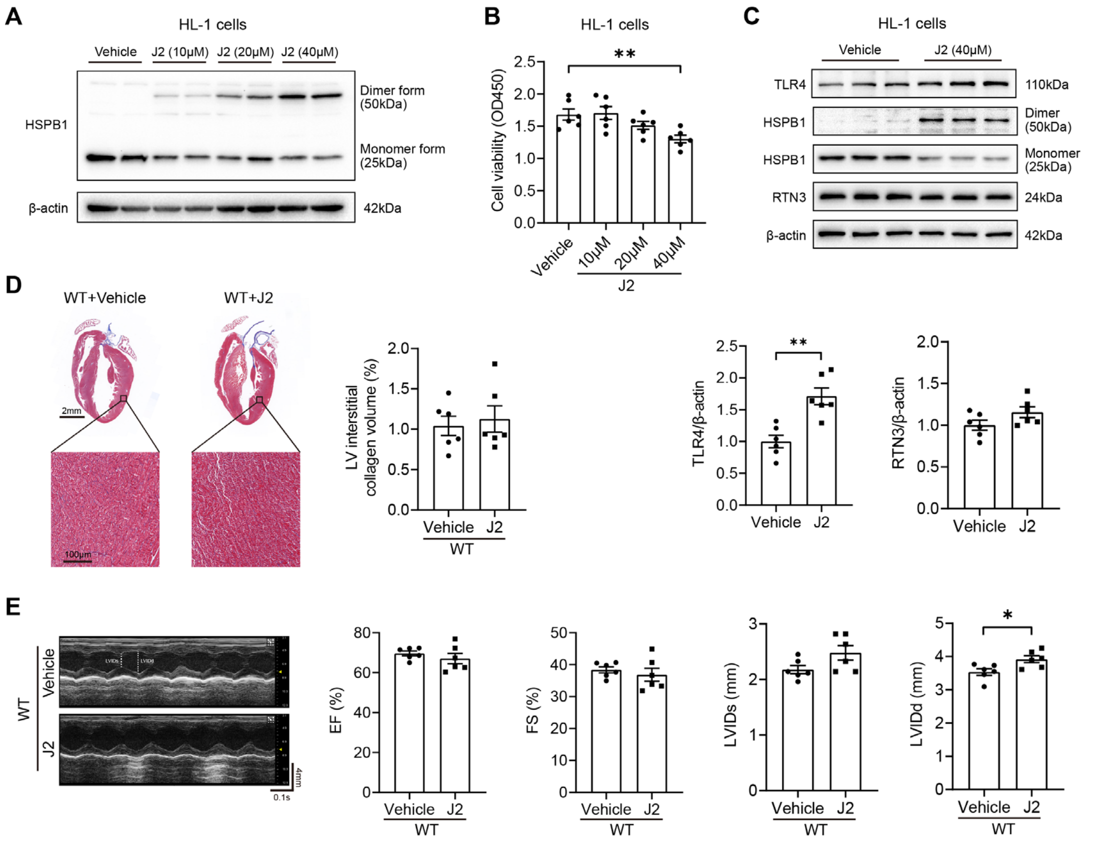

Figure S7. J2 treatment reduced the HSPB1 monomer form but had no significant effect on cardiac ejection function in wild-type mice.

(A) Representative western blots of HSPB1 different forms in HL-1 cells treated with J2. (B) HL-1 cells were treated with different concentrations of J2, and CCK-8 was used to determine cell viability (n=6 per group). (C) Western blot analysis of TLR4 and RTN3 expression in HL-1 cells treated with J2 (n=6 per group). (D) Representative Masson trichrome staining images and quantification of collagen volume after mice were injected with J2 for 4 consecutive weeks (n=6 per group). (E) Representative M-mode echocardiography images and parameters, including EF, FS, LVIDs, and LVIDd (n=6 per group). Data are presented as mean ± SEM. Data were analyzed by unpaired Student's *t* test. **P*<0.05, ***P*<0.01.

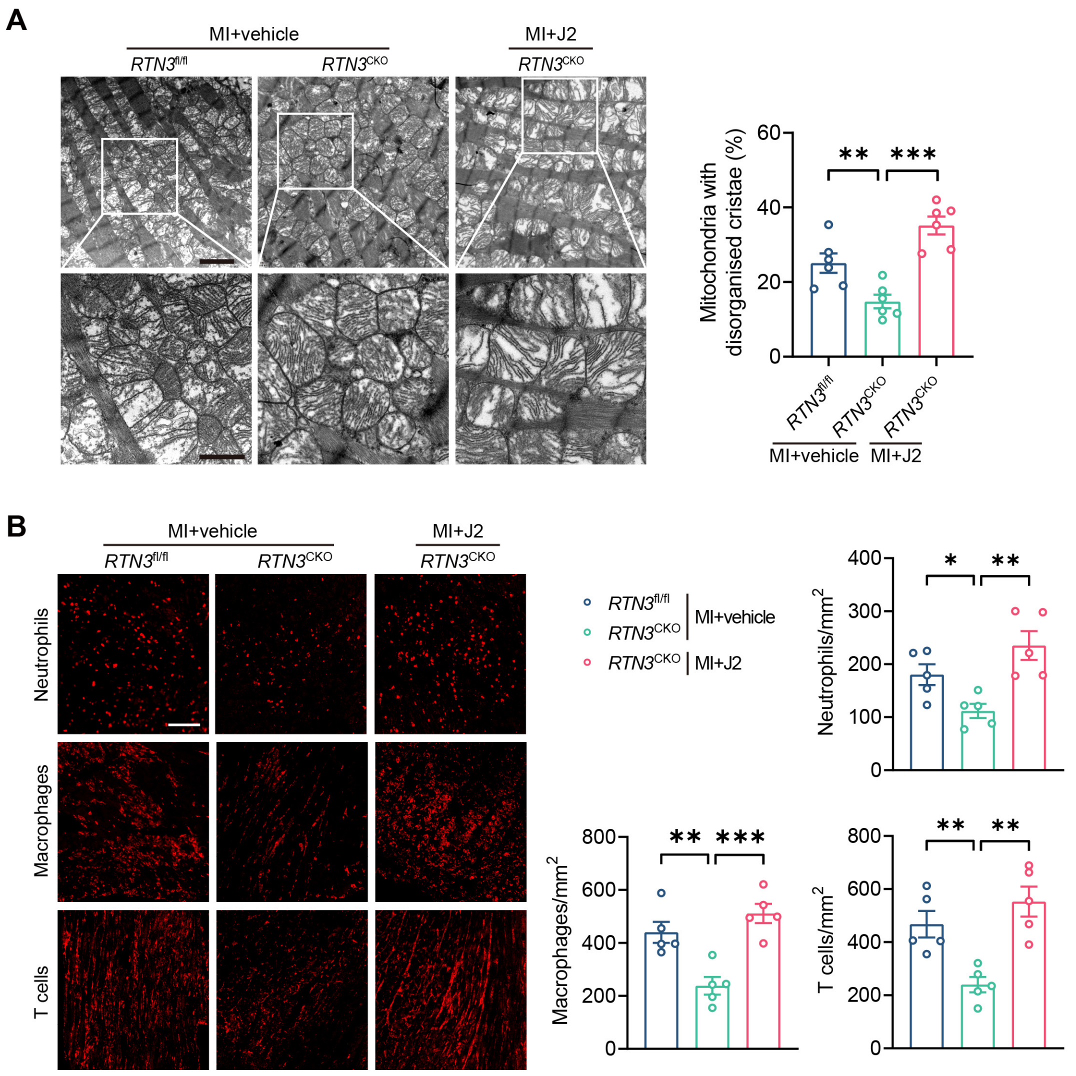

Figure S8. HSPB1 inhibition eliminates the protective effect of *RTN3* knockout on mitochondrial morphological abnormalities and immune cell infiltration after MI. (A) Representative transmission electron microscopy images and quantification of abnormal mitochondria in MI mice treated with J2 (n=6 per group). The upper scale bar=2 μm, and the lower scale bar=1 μm. (B) Representative immunofluorescence images and quantitative analysis of immune cell infiltration in indicated groups (n=5 per group). Scale bar=100 μm. Ly6G, F4/80, and CD3 were used as markers for neutrophils, macrophages and T cells, respectively. Data are presented as mean ± SEM. Statistical significance was assessed by one-way ANOVA with a Bonferroni post hoc test. **P*<0.05, ***P*<0.01, ****P*<0.001.

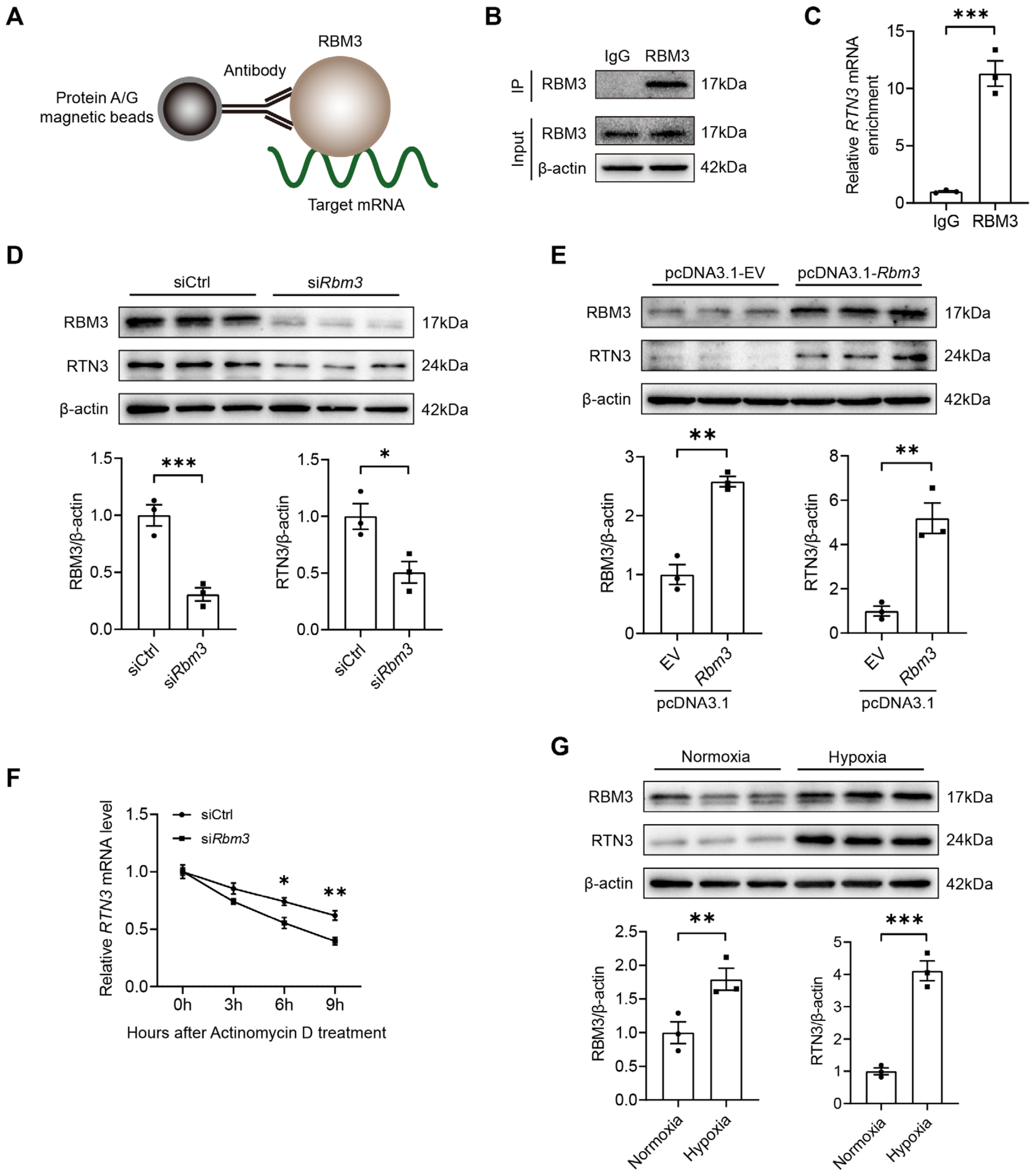

Figure S9. RBM3 positively regulates RTN3 protein expression by binding to and stabilizing its mRNA.

(A) Schematic diagram of an RNA immunoprecipitation (RIP) assay. (B) Representative western blots of IP efficiency using anti-RBM3 antibodies in NRCMs. (C) Relative mRNA level of *RTN3* was measured on RIP samples. (D) Representative western blots and quantification of RBM3 and RTN3 in H9c2 cells transfected with indicated siRNA (n=3 per group). (E) Representative western blots and quantification of RBM3 and RTN3 in H9c2 cells transfected with indicated plasmid (n=3 per group). (F) The stability of *RTN3* mRNA in H9c2 cells was measured at indicated time points after actinomycin D treatment (n=5 per group). (G) Representative western blots and

quantification of RBM3 and RTN3 in NRCMs subjected to hypoxia for 6 hours (n=3 per group). Data are presented as mean ± SEM. Statistical significance was assessed by unpaired Student's *t* test. **P*<0.05, ***P*<0.01, ****P*<0.001.

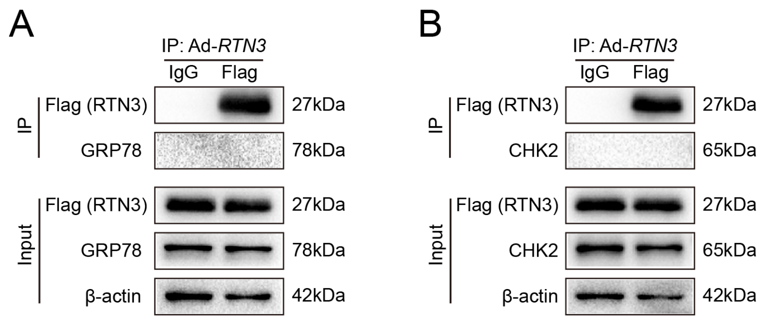

Figure S10. Validation of the interaction between RTN3 and other proteins in NRCMs.

(A) Co-IP experiment to verify whether RTN3 interacts with GRP78. (B) Co-IP experiment to verify whether RTN3 interacts with CHK2.

Supplemental Tables

Table S1. Clinical data in patients with AMI on the first day after PCI.

Characteristics	Patients with low RTN3（n=46）	Patients with high RTN3（n=45）	*P* values
Age（years）	62.13±1.90	60.53±1.54	0.516
Males, n (%)	39 (85%)	39 (87%)	0.797
BMI（kg/m^2）	24.64±0.39	25.14±0.37	0.352
Systolic blood pressure (mmHg)	134.80±3.52	133.20±2.92	0.731
Diastolic blood pressure (mmHg)	80.02±1.87	83.44±2.36	0.257
History of hypertension, n (%)	26（57%）	28（62%）	0.580
History of diabetes, n (%)	6（13%）	11（24%）	0.163
History of smoking, n (%)	19（41%）	21（47%）	0.606
FBG (mmol/L)	6.38±0.32	6.97±0.48	0.299
TC (mmol/L)	4.31±0.18	4.09±0.14	0.330
TG (mmol/L)	1.68±0.15	1.58±0.14	0.617
HDL-C (mmol/L）	0.96±0.04	0.96±0.03	0.923
LDL-C (mmol/L)	2.61±0.16	2.33±0.11	0.155
ALT (U/L)	31.63±1.92	38.53±4.66	0.171
AST (U/L)	97.33±17.59	121.30±25.79	0.443
Cr (μmol/L)	82.96±14.63	86.76±17.28	0.867
BUN (mmol/L)	5.46±0.48	5.52±0.33	0.922
GFR (mL/min)	92.06±3.71	94.52±3.62	0.636
Culprit vessel			
LAD	24（52%）	26（58%）	0.591
LCX	14（30%）	16（36%）	0.603
RCA	15（33%）	16（36%）	0.769
Stent number implanted	1.62±0.19	1.40±0.14	0.343
STEMI, n (%)	23（50%）	25（56%）	0.596

Data are presented as mean ± SEM or number (percentages). Student's *t* test and Chi-square test were used for statistical analysis. BMI: body mass index; FBG: fasting blood glucose; TC: total cholesterol; TG: triglycerides; HDL-C: high density lipoprotein cholesterol; LDL-C: low density lipoprotein cholesterol; ALT: alanine aminotransferase; AST: aspartate aminotransferase; Cr: creatinine; BUN: blood urea nitrogen; GRF: glomerular filtration rate; LAD: left anterior descending coronary artery; LCX: left circumflex coronary artery; RCA: right coronary artery; STEMI: ST-segment elevation myocardial infarction.

Table S2. Clinical data in patients with AMI in the sixth month after PCI.

Characteristics	Patients with low RTN3（n=29）	Patients with high RTN3（n=29）	*P* values
Age（years）	61.00±1.91	59.92±2.01	0.703
Males, n (%)	21 (72%)	22 (76%)	>0.99
BMI（kg/m^2）	23.75±0.27	23.39±0.34	0.397
Systolic blood pressure (mmHg)	121.60±1.59	124.90±2.91	0.286
Diastolic blood pressure (mmHg)	70.29±1.28	69.21±1.83	0.618
History of hypertension, n (%)	18（62%）	20（69%）	0.783
History of diabetes, n (%)	5（17%）	4（14%）	>0.99
History of smoking, n (%)	9（31%）	11（38%）	0.783
FBG (mmol/L)	5.49±0.10	5.65±0.22	0.473
TC (mmol/L)	4.31±0.20	4.38±0.27	0.827
TG (mmol/L)	1.48±0.14	1.47±0.12	0.952
HDL-C (mmol/L）	0.99±0.06	0.89±0.03	0.240
LDL-C (mmol/L)	2.47±0.18	2.52±0.22	0.864
ALT (U/L)	28.88±3.80	28.05±3.50	0.878
AST (U/L)	28.17±2.18	26.24±1.45	0.506
Cr (μmol/L)	71.28±2.92	71.33±3.36	0.991
BUN (mmol/L)	5.08±0.24	5.76±0.45	0.158
GFR (mL/min)	92.68±2.79	95.97±3.41	0.459
Culprit vessel			
LAD	18（62%）	19（66%）	>0.99
LCX	8（28%）	9（31%）	>0.99
RCA	9（31%）	9（31%）	>0.99
Stent number implanted	1.66±0.14	1.75±0.21	0.701
STEMI, n (%)	14（48%）	15（52%）	>0.99

Data are presented as mean ± SEM or number (percentages). Student's t test and Fisher's exact test were used for statistical analysis. BMI: body mass index; FBG: fasting blood glucose; TC: total cholesterol; TG: triglycerides; HDL-C: high density lipoprotein cholesterol; LDL-C: low density lipoprotein cholesterol; ALT: alanine aminotransferase; AST: aspartate aminotransferase; Cr: creatinine; BUN: blood urea nitrogen; GRF: glomerular filtration rate; LAD: left anterior descending coronary artery; LCX: left circumflex coronary artery; RCA: right coronary artery; STEMI: ST-segment elevation myocardial infarction.

Table S3. Clinical data in control subjects and MI patients.

Characteristics	Con（n=8）	MI（n=16）	*P* values
Age（years）	60.50±3.77	62.81±2.81	0.634
Males, n (%)	7 (88%)	14 (88%)	>0.99
BMI（kg/m^2）	25.00±1.02	24.35±0.75	0.620
Systolic blood pressure (mmHg)	126.40±6.53	129.30±4.65	0.724
Diastolic blood pressure (mmHg)	72.50±4.19	75.06±2.91	0.618
History of hypertension, n (%)	4（50%）	9（56%）	>0.99
History of diabetes, n (%)	1（13%）	2（13%）	>0.99
History of smoking, n (%)	5（63%）	6（38%）	0.391
FBG (mmol/L)	6.25±0.43	6.13±0.35	0.839
TC (mmol/L)	4.20±0.23	4.12±0.25	0.830
TG (mmol/L)	1.71±0.13	1.61±0.28	0.795
HDL-C (mmol/L）	0.96±0.03	0.99±0.07	0.749
LDL-C (mmol/L)	2.58±0.28	2.44±0.22	0.704
ALT (U/L)	29.00±2.50	28.88±2.22	0.972
AST (U/L)	50.50±8.73	50.31±7.70	0.988
Cr (μmol/L)	68.00±3.79	62.35±3.49	0.327
BUN (mmol/L)	4.98±0.36	5.19±0.38	0.720
GFR (mL/min)	97.32±7.41	99.32±3.35	0.778

Data are presented as mean ± SEM or number (percentages). Student's *t* test and Fisher's exact test were used for statistical analysis. BMI: body mass index; FBG: fasting blood glucose; TC: total cholesterol; TG: triglycerides; HDL-C: high density lipoprotein cholesterol; LDL-C: low density lipoprotein cholesterol; ALT: alanine aminotransferase; AST: aspartate aminotransferase; Cr: creatinine; BUN: blood urea nitrogen; GRF: glomerular filtration rate.

Table S4. **Antibodies, siRNA, and oligonucleotides sequences.**

Antibodies	Source	Identifier
RTN3	Abcam	Cat# ab187764; RRID: AB_2922981
RTN3	Santa Cruz	Cat# sc-374599; RRID: AB_10986405
Flag	Cell Signaling Technology	Cat# 14793; RRID: AB_2572291
Flag	Sigma-Aldrich	Cat# F1804; RRID: AB_262044
Myc	Proteintech	Cat# 16286-1-AP; RRID: AB_11182162
cTnT	Proteintech	Cat# 26592-1-AP; RRID: AB_2880566
Ndufs1	Proteintech	Cat# 12444-1-AP; RRID: AB_2282657
Atp5a	Proteintech	Cat# 14676-1-AP; RRID: AB_2061761
Uqcrc2	Proteintech	Cat# 14742-1-AP; RRID: AB_2241442
Sdhb	Proteintech	Cat# 10620-1-AP; RRID: AB_2285522
Cox5a	Proteintech	Cat# 11448-1-AP; RRID: AB_2085429
HSPB1	Proteintech	Cat# 18284-1-AP; RRID: AB_2295540
HSPB1	Cell Signaling Technology	Cat# 2442; RRID: AB_2233273
Calreticulin	Proteintech	Cat# 27298-1-AP; RRID: AB_2880835
Histone-H3	Proteintech	Cat# 17168-1-AP; RRID: AB_2716755
TLR4	Proteintech	Cat# 66350-1-Ig; RRID: AB_2881730
PGC1α	Proteintech	Cat# 66369-1-Ig; RRID: AB_2828002
NFκB	Cell Signaling Technology	Cat# 8242; RRID: AB_10859369
Phospho-NFκB	Cell Signaling Technology	Cat# 3033; RRID: AB_331284
IκBα	Cell Signaling Technology	Cat# 4814; RRID: AB_390781
Phospho-IκBα	Cell Signaling Technology	Cat# 9246; RRID: AB_2267145
RBM3	Proteintech	Cat# 14363-1-AP; RRID: AB_2269266
Ly6G	Proteintech	Cat# 65140-1-Ig; RRID: AB_2881475
F4/80	Proteintech	Cat# 28463-1-AP; RRID: AB_2881149
CD3	Proteintech	Cat# 17617-1-AP; RRID: AB_1939430
β-actin	Proteintech	Cat# 20536-1-AP; RRID: AB_10700003
GAPDH	Proteintech	Cat# 10494-1-AP; RRID: AB_2263076
Rabbit IgG	Cell Signaling Technology	Cat# 2729; RRID: AB_1031062
Mouse IgG	Cell Signaling Technology	Cat# 5415; RRID: AB_10829607
**siRNA**		
Tlr4 siRNA	Tsingke Biotechnology	5'-GGCUCAUAAUCUUAUACAU-3'
Hspb1 siRNA	Tsingke Biotechnology	5'-CGAGGCCCGUGCCCAAAUU-3'
si*Rbm3* siRNA	Tsingke Biotechnology	5'-GUUUGUUAAAGAACUUUAAGA-3'
**Oligonucleotides**		
RTN1 (Human)	Sangon Biotech	F: GTGCAGAAAACCGACGAAGG<br>R: GCAGGCAGTCCGTGTACTT
RTN2 (Human)	Sangon Biotech	F: CTTTAGCATCGTGTCCGTGG<br>R: CTTTGCGGTAAACCCTGAGAG
RTN3 (Human)	Sangon Biotech	F: ACTGGGTTTGTCTTTGGCAC<br>R: ATGACGGACTTGTAGATCCTGA

RTN4 (Human)	Sangon Biotech	F: GTTGACCTCCTGTACTGGAGA<br>R: CTGTTACGCTCACAATGCTGA
β-actin (Human)	Sangon Biotech	F: CCTGGCACCCAGCACAAT<br>R: GGGCCGGACTCGTCATAC
RTN3 (Rat)	Sangon Biotech	F: AGTCTCCGTCAGTCTCCTCG<br>R: ATCAGATCATGCACCGCACA
β-actin (Rat)	Sangon Biotech	F: TGTCACCAACTGGGACGATA<br>R: GGGGTGTTGAAGGTCTCAAA
RTN3 (Mouse)	Sangon Biotech	F: TCGTGTGCGGTGCACGAT<br>R: TTGTTGACATGCACCATCGC
ANP (Mouse)	Sangon Biotech	F: GCTTCCAGGCCATATTGGAGCA<br>R: TCTCTCAGAGGTGGGTTGACCT
BNP (Mouse)	Sangon Biotech	F: ATGGATCTCCTGAAGGTGCTGT<br>R: GCAGCTTGAGATATGTGTCACC
Col1a1 (Mouse)	Sangon Biotech	F: GGAGACAGGTCAGACCTGTGTG<br>R: CAGCTGGATAGCGACATCGGC
Col3a1 (Mouse)	Sangon Biotech	F: ATATCAAACACGCAAGGC<br>R: ATTAAAGCAAGAGGAACAC
Ndufa1 (Mouse)	Sangon Biotech	F: CCGGAAGAGAGGTAAAGCCG<br>R: ACATCTCCGCACCGTTACTC
Ndufc1 (Mouse)	Sangon Biotech	F: AAGTTCTATGTCCGGGAGCC<br>R: CCAACTGCCAACCAGTTAGGT
Ndufs1 (Mouse)	Sangon Biotech	F: TGCAAATCCCTCGATTCTGTTAC<br>R: GCTTTCTCAATCTCTACCAGGC
Sdha (Mouse)	Sangon Biotech	F: GGAACACTCCAAAAACAGACCT<br>R: CCACCACTGGGTATTGAGTAGAA
Sdhb (Mouse)	Sangon Biotech	F: ATTTACCGATGGGACCCAGAC<br>R: GTCCGCACTTATTCAGATCCAC
Sdhd (Mouse)	Sangon Biotech	F: TGGTCAGACCCGCTTATGTG<br>R: GGTCCAGTGGAGAGATGCAG
Uqcrb (Mouse)	Sangon Biotech	F: GGCCGATCTGCTGTTTCAG<br>R: CATCTCGCATTAACCCCAGTT
Uqcrc1 (Mouse)	Sangon Biotech	F: AGACCCAGGTCAGCATCTTG<br>R: GCCGATTCTTTGTTCCCTTGA
Uqcrh (Mouse)	Sangon Biotech	F: GTGGACCCCCTAACAACAGTG<br>R: CGGGAAGACACGCGATTATCA
Cox5a (Mouse)	Sangon Biotech	F: GCCGCTGTCTGTTCCATTC<br>R: CCAGGCATCAATGTCTGGCT
Cox6a2 (Mouse)	Sangon Biotech	F: CTGCTCCCTTAACTGCTGGAT<br>R: GATTGTGGAAAAGCGTGTGGT
Cox7a1 (Mouse)	Sangon Biotech	F: GCTCTGGTCCGGTCTTTTAGC<br>R: GTACTGGGAGGTCATTGTCGG
Atp5a1 (Mouse)	Sangon Biotech	F: TCTCCATGCCTCTAACACTCG<br>R: CCAGGTCAACAGACGTGTCAG

Atp5g1 (Mouse)	Sangon Biotech	F: CGCTCAGACCAAGGGCTAAA<br>R: CCCTGGTACAGGAGCGAATC
Atp5k (Mouse)	Sangon Biotech	F: GGAGAGGAGAATAGCAGCGG<br>R: ATCTTGAGCTTCCGCCAGTT
IL-1β(Mouse)	Sangon Biotech	F: TCTTCCTAAAGTATGGGCTGGA<br>R: AAAGGGAGCTCCTTAACATGC
IL-6 (Mouse)	Sangon Biotech	F: CAAAGCCAGAGTCCTTCAGAG<br>R: GCCACTCCTTCTGTGACTCC
IL-10 (Mouse)	Sangon Biotech	F: ATCGATTTCTTCCCTGTGAA<br>R: TGGCCTTGTAGACACCTTGG
Arg1 (Mouse)	Sangon Biotech	F: GAATCTGCATGGGCAACC<br>R: GAATCCTGGTACATCTGGGAAC
β-actin (Mouse)	Sangon Biotech	F: GTGCTATGTTGCTCTAGACTTCG<br>R: ATGCCACAGGATTCCATACC
